# Exploring the anti-anaphylaxis potential of natural products: A Review

**DOI:** 10.1007/s10787-025-01685-2

**Published:** 2025-03-19

**Authors:** Aya H. Eid, Eman S. Zaki, Miral O. Sabry, Riham A. El-Shiekh, Samar S. Khalaf

**Affiliations:** 1 Department of Pharmacology and Toxicology, Faculty of Pharmacy, Heliopolis University, Cairo, Egypt; 2 Faculty of Science, National University of Singapore, Singapore, Singapore; 3 Institute of Manufacturing Technology (SIMTech), Agency for Science, Technology and Research (A*STAR), Singapore, Singapore; 4 Department of Pharmacognosy, Faculty of Pharmacy, Cairo University, Kasr El-Aini Street, Cairo, 11562 Egypt; 5 Biochemistry Department Faculty of Pharmacy, Heliopolis University, Cairo, Egypt

**Keywords:** Natural products, Mast cells, Anaphylaxis, Allergy, Inflammatory mediators

## Abstract

Allergies are a common health issue affecting many people around the world, especially in developed countries. They occur when the immune system overreacts to substances that are usually harmless. Some common allergic conditions include asthma, sinus infections, skin rashes, food allergies, hay fever, severe allergic reactions, eczema, swelling, and reactions to medications or insect stings. The causes of these allergies are complex and often linked to genetics, which can lead to heightened immune responses known as atopy. Throughout history, plant extracts have been used for various purposes, including medicine and food. In addition, their bioactive compounds show a wide range of beneficial effects, such as reducing allergic reactions, fighting oxidative stress, mast cell stabilizers, and lowering inflammation, highlighting their potential for treating various health conditions. Flavonoids and phenolic compounds are commonly used in anaphylaxis for their potent anti-inflammatory action. This review aims to promote the use of natural products as potential treatments for anaphylaxis. In addition, the discovery of new drugs derived from natural sources holds significant promise for the management of anaphylaxis.

## Introduction

Anaphylaxis is a serious and potentially life-threatening allergic reaction that poses a significant challenge for individuals who are sensitive to allergens, as well as for their caregivers. Characterized by its sudden onset and rapid progression, anaphylaxis requires prompt recognition, swift intervention, and meticulous management to reduce its severe consequences (Karunarathna et al. 2024). Anaphylaxis can be triggered by a variety of allergens, including foods, insect stings, and medications (Worm et al. 2014). The pathophysiology of anaphylaxis primarily involves immunoglobulin E (IgE) antibodies that bind to specific allergens. This binding activates mast cells and basophils, leading to the release of inflammatory mediators such as histamine, leukotrienes, and cytokines. These mediators cause various physiological effects, including bronchoconstriction, vasodilation, increased vascular permeability, and ultimately result in symptoms such as difficulty breathing, skin flushing, hives, and a drop in blood pressure (Cingi et al. 2020).

Where the serious nature of anaphylaxis and its potential for rapid progression to life-threatening conditions are concerned, immediate recognition and intervention are crucial. Continued research into the mechanisms underlying anaphylaxis and the identification of effective treatments is essential for improving patient outcomes. Given the high prevalence and limitations of current treatments for anaphylaxis, there is an urgent need to discover new effective therapies. In recent decades, natural products have emerged as key resources for drug discovery due to their structural and chemical diversity, as well as their wide range of biological activities (Farhan et al. 2024; Liu et al. 2024). Numerous studies have demonstrated that natural products possess significant pharmacological properties, including immunomodulatory, anti-allergic, anti-inflammatory, and antioxidant effects (Chen et al. 2020; Farhan et al. 2024; Fonacier et al. 2024). These findings have spurred interest in the investigation and application of natural products for treating allergic diseases. However, there is a notable lack of reviews addressing the progress of natural products in anaphylaxis management. This review aims to highlight recent advancements in understanding the emerging pathogenesis of anaphylaxis. In addition, we provide a summary of the therapeutic effects and underlying mechanisms of natural phytochemicals, mainly polyphenolics, on anaphylaxis, along with pathophysiology and current treatments.

## Causes and triggers of anaphylactic reaction

Anaphylaxis may be caused by several allergies, including specific foods, drugs, latex, and insect bites. The European Anaphylaxis Registry indicates that food, particularly eggs, cow’s milk, and nuts, is the primary trigger of anaphylaxis in children and adolescents (DuToit et al. 2024). Reactions to insect venom have also been observed in young adulthood. Although reactions to the venoms of snakes, other invertebrates, and cold-blooded vertebrates have also been documented, hymenopteran (bee and wasp) stings are the most common cause in North America and Europe (Stevens et al. 2023).

Important factors precipitating anaphylaxis involve not only allergen-related ones including its kind, amount, and physical and chemical stability, but also patient-related (endogenous) and environmental (exogenous) factors (Rossi et al. 2022; Cardona et al. 2020). Identifying the causes and risk factors for anaphylaxis is critical for preventing recurrence and educating patients on how to avoid known allergen exposures (Cimen et al. 2023).

According to European Registry research, the main risk factor is becoming older, and this seems to be linked to increased mast cell degranulation (Worm et al. 2018). Female sex has historically been thought to be a risk factor for food allergies and asthma as a result of enhanced mast cell activation and allergic sensitization caused by biological sex-specific hormones. Nevertheless, recent research has shown that anaphylaxis in both adults and children is more often caused by male sex (Pastorello et al. 2021).

Furthermore, the clinical progression and risk factors for anaphylaxis in children may differ from those in adults. Recent globalization has augmented the number of potential triggers, especially due to the worldwide distribution of food, the advancement of novel pharmaceutical substances, and the impact of climate change on regional insect populations. In adults, the most often reported triggering stimuli include medicines (35%), food (32%), insect venom (19%), and idiopathic (14%). In children, the predominant causes are associated with food (85%), followed by idiopathic (11%) and insect venom (4%) (Manchanda & Das 2023).

Pharmaceutical interventions such as antibiotics, monoclonal antibodies, non-steroidal anti-inflammatory medicines (NSAIDs), chemotherapeutic agents, and radiocontrast media are among the most often linked pharmaceuticals with anaphylaxis. Common perioperative triggers include neuromuscular blocking drugs, opioids, antibiotics, latex, and blood product transfusions (Regateiro et al. 2020).

Cofactors that exacerbate clinical symptoms and elevate the likelihood of poor consequences encompass infectious diseases, exercise, underlying mast cell disorders, stress, active allergic conditions such as asthma, advanced age, consumption of specific medications, delayed or omitted administration of adrenaline, and a history of prior anaphylaxis (DuToit et al. 2024).

## Clinical presentation and symptoms

The presenting features of anaphylaxis can vary among patients, influenced by factors such as the triggering agent, timing of presentation, patient age, co-morbid conditions, and concurrent medication use (Dribin et al. 2021). Anaphylaxis signs and symptoms generally appear within minutes to 2 h following allergen exposure, food allergens frequently provoke symptoms within 30 min, while drug and insect stings induce more rapid reactions. Delayed anaphylaxis responses (occur within 3 to 5 h) following the consumption of red mead have recently been reported in patients with IgE specific for galactose alpha-1,3-galactose (alpha-gal) (Comberiati et al. 2024).

Anaphylaxis can impact multiple organ systems, primarily through the secretion of mediators from mast cells and basophils. This is particularly applicable to the tissues of the skin, pulmonary system, cardiac system, central nervous system, and gastrointestinal system. The circulatory system is significantly engaged during moderate anaphylactic reactions and is crucial in severe instances, where anaphylactic shock may occur (Martínez-Fernandez et al. 2019).

Approximately, 90% of the individuals are estimated to develop cutaneous symptoms, which may include urticaria, angioedema, flushing, and pus. Dermatological manifestations in a patient suspected of anaphylaxis serve as a useful, but not definitive, diagnostic indicator. Multiple organ systems may be concurrently involved. Atypical symptoms, including substernal chest pain, headache, or seizure, may infrequently manifest during anaphylaxis. The variability in anaphylaxis presentation can complicate diagnosis (Soucy and Michaud 2023). Common symptoms of anaphylaxis reaction are presented in Table 1 (Tomasiak-Łozowska et al. 2018).

**Table 1:** Common symptoms of anaphylaxis reaction

Skin (80–90%)	Itching, conjunctival congestion, maculopapular rash, angioedema, lip, tongue swelling, erythema, and urticaria
Respiratory system (70%)	Rhinorrhea, nasal itching, wheezing, stridor, sneezing, cough, dyspnea, throat tightness, tachypnea, and respiratory arrest
Gastrointestinal system (45%)	Pain in abdomen, difficulty swallowing, nausea, diarrhea, and vomiting
Central nervous system (15%)	Confusion, altered mental status, dizziness, tubular vision, and headache
Cardiovascular system (45%)	Arrhythmias, chest pain/tightness, cardiac arrest, hypotension, shock, and palpitations

## Pathophysiology

Anaphylaxis is generally a multiorgan event that engages various effector cells, such as mast cells, basophils, neutrophils, macrophages, and platelets. Anaphylaxis can be classified mechanistically into three categories: immunologic, nonimmunologic, and idiopathic (Bilò et al. 2021).

The idiopathic category is characterized by an unknown allergen or underlying mastocytosis, a clonal mast cell disorder. Approximately, 30–60% of the patients experiencing anaphylaxis may lack an obvious etiological trigger, leading to the classification of their condition as idiopathic anaphylaxis, which is ultimately a diagnosis of exclusion. Recently, expressions such as anaphylactoid and pseudo-allergic responses have gradually been supplanted with mechanism-based descriptions (Martínez-Fernandez et al. 2019; Nguyen et al. 2021).

Immunologic anaphylaxis can be classified into immunoglobulin E (IgE)-mediated forms, such as those triggered by food, drugs, and insect stings, and IgE-independent forms, which encompass immunoglobulin G (IgG)-dependent anaphylaxis, exemplified by high molecular weight iron dextran and the infusion of human monoclonal antibodies like infliximab, as well as complement-mediated anaphylaxis, including over sulfated chondroitin sulfate-contaminated heparin and polyethylene glycols. Chemotherapy can elicit mixed responses involving both IgE- and non-IgE-mediated mechanisms (Dribin et al. 2023).

### Immunoglobulin E-mediated pathway

**Fig. 1:**
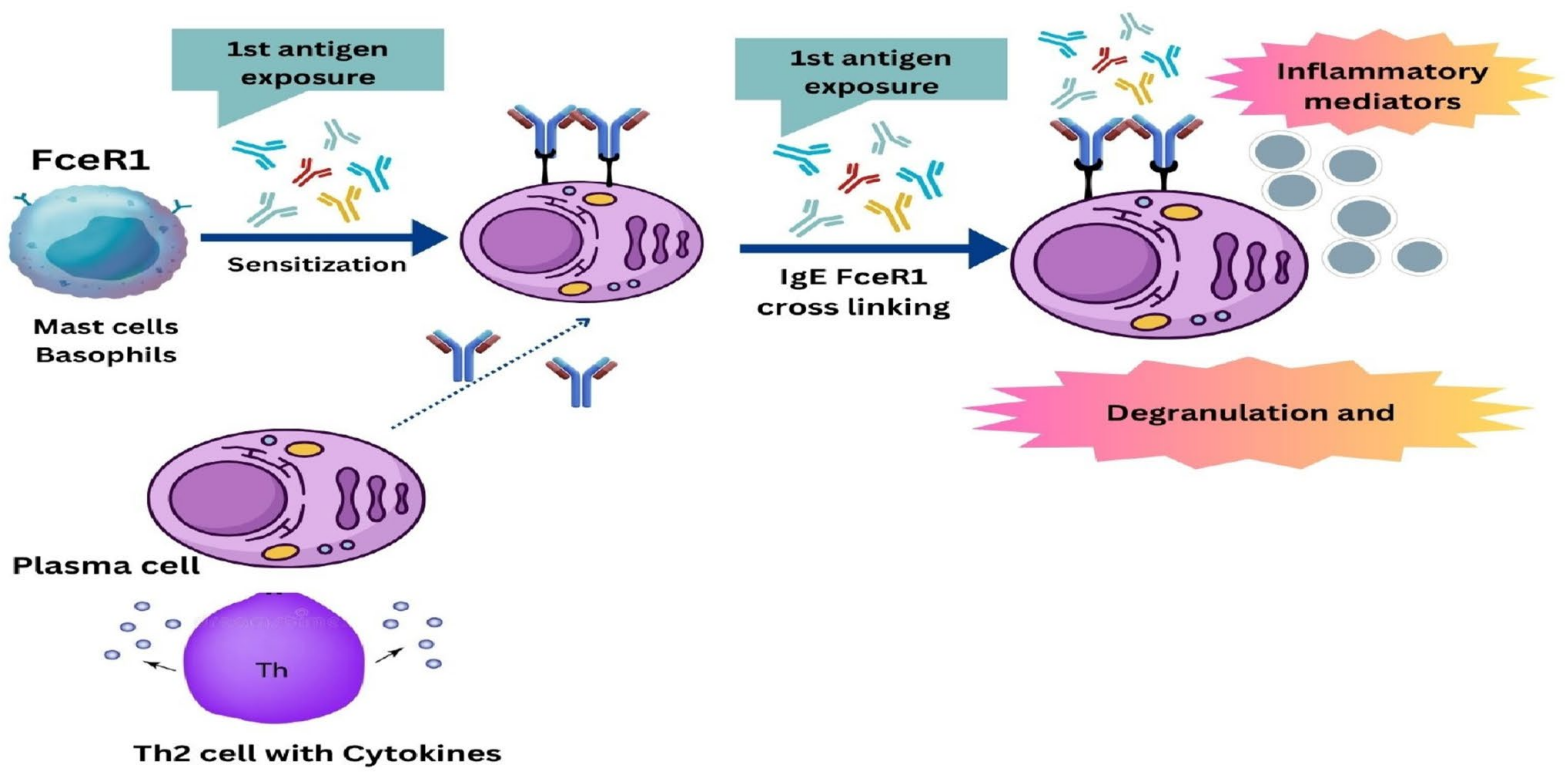
IgE-mediated anaphylaxis

Reactions mediated by IgE to allergens are a recognized cause of anaphylaxis. These reactions entail the binding of IgE to the FceR1 receptor present on the surface of basophils and mast cells. Furthermore, exposure to a bi- or multivalent allergen leads to the cross-linking of Fcer1-bound IgE, which activates mast cells and basophils (Krishnaswamy 2021). This induces the immediate release of stored chemical mediators and also results in the de novo synthesis of additional inflammatory mediators as illustrated by Fig. 1. The chemical mediators include lipoxygenases, tryptase, carboxypeptidase A, proteoglycans, and histamine. Through the activation of cyclooxygenases, phospholipase A, and arachidonic acid, metabolites are produced which include leukotrienes, platelet-activating factors and prostaglandins (Jimenez-Rodriguez et al. 2018; Valenta et al. 2018).

The inflammatory response is subsequently regulated by tumor necrosis factor (TNF-alpha), a late-phase reactant that has already been produced. Detailed explanations of the physiology of these chemical mediators are provided as follows (McLendon & Sternard 2024):

- Histamine enhances vascular permeability and induces vasodilation, resulting in tissue hypoperfusion. The body reacts to these alterations by elevating heart rate and enhancing cardiac contraction.
- Prostaglandin D acts as a bronchoconstrictor, concurrently restricting cardiac and pulmonary arteries. It also enhances peripheral vasodilation, leading to the hypoperfusion of essential organs.
- Leukotrienes contribute to bronchoconstriction, increase vascular permeability, and promote airway remodeling.
- The platelet activation factor functions as a bronchoconstrictor and enhances vascular permeability TNF-alpha stimulates neutrophils, contributing to stress-induced leukocytosis, and enhances chemokine production.

Nonetheless, IgE levels alone are insufficient to account for an individual’s risk of developing anaphylaxis. Individuals exhibiting nearly undetectable levels of specific IgE may still undergo near-fatal anaphylaxis. On the other hand, certain patients may not exhibit allergic reactions despite the presence of elevated levels of allergen-specific antibodies upon exposure to the allergen. Consequently, an alternative IgE-independent pathway for anaphylaxis must exist (Ebo et al. 2019) (Nuñez-Borque et al. 2022).

### Immunoglobulin G-mediated pathway (IgE-independent pathway)

While allergies and anaphylaxis are traditionally attributed to IgE antibodies in humans, there is growing evidence that, in specific situations, IgG-dependent mechanisms may also play a role in these responses (Godon et al. 2021). Research conducted on humans and animal studies indicate that IgG, whether alone or in immune complexes, is capable of activating mast cells and either initiate or exacerbate the severity of anaphylactic reactions. IgG antibodies interact with Fc gamma receptors (FcgRs), which exhibit varying affinities and are present on various cell types (Cianferoni 2021).

Among numerous receptor categories implicated in activation signaling, FcgRI is the sole receptor that exhibits high-affinity binding to monomeric IgG, specifically IgG1 and IgG3. It is expressed on both mast cells and neutrophils. FcgRI binding to particular IgG1 may induce mast cell activation. Both IgG1 and IgG3 are capable of activating FcgRI present on macrophages and monocytes. While FcgRI receptors are often bound by monomeric IgG, this does not inhibit their activation by IgG immune complexes. The latter has greater binding affinity and can thus displace monomeric IgG, so eliciting hypersensitivity responses (Kow et al. 2019).

Mouse models’ researchers demonstrate that antigens can stimulate basophils, neutrophils, and macrophages to release platelet-activating factors (PAF) through the activation of FcgRIII or FcgRIV. These models indicate that IgG necessitates elevated levels of specific IgG antibodies and antigens, unlike IgE-mediated anaphylaxis. This is probably attributable to the reduced affinity of FcgRs in comparison to FcεRI (Fowler & Lieberman 2022).

Furthermore, murine studies have shown that blocking IgG response may be induced by low-dose antigens, which can prevent IgE-mediated anaphylaxis. Conversely, large concentrations of antigens have the potential to induce IgG-mediated anaphylaxis (Jimenez-Rodriguez et al. 2018). Possible instances of IgG-mediated anaphylaxis encompass reactions to the infusion of biological agents such as dextrans, infliximab, aprotinin, intravenous immunoglobulin in individuals with IgA deficiency, Von Willebrand factor infusions, along with various other medicines (Jimenez-Rodriguez et al. 2018; Jönsson et al. 2019).

### Complement activation pathway

The ultimate pathway associated with the immunological mechanisms underlying anaphylaxis is the activation of complement. The start of the complement cascade is triggered by a variety of stimuli, leading to the production of C3a, C4a, and C5a. The mediators are known as anaphylatoxins (Fowler & Lieberman 2022). A multitude of evidence indicates the synthesis of C3a and C5a, alongside the reduction of complement levels in instances of human anaphylaxis. This indicates that these anaphylatoxins may contribute to the occurrence of anaphylaxis. Upon the activation of the complement system, the release of C3a and C5a occurs, which in turn initiates the activation of basophils, endothelial cells, mast cells, and smooth muscle cells through their specific receptors (Stevens et al. 2023).

## How to manage anaphylaxis

### Triage

Allergic reactions demand prompt triage due to the potential for rapid escalation to anaphylaxis, a life-threatening condition. Early identification and timely intervention are pivotal in mitigating severe consequences. Patients experiencing anaphylaxis must be urgently transported to a medical facility, where comprehensive care begins even during ambulance transit. Upon hospital admission, continuous monitoring of vital parameters, including electrocardiogram, pulse, and blood pressure, is essential to assess the severity of the reaction, detect shock, and evaluate the risk of biphasic anaphylactic responses (Bock et al. 2007; Carter et al. 2020).

### Airway management

Establishing and maintaining a patent airway is the paramount priority in managing anaphylaxis. Clinical indicators of airway compromise are perioral edema, angioedema, and stridor. In cases of impending airway compromise, immediate definitive airway management, including endotracheal intubation, is critical to avert a failed intubation due to progressive edema. Delay increases the likelihood of requiring emergent surgical interventions, such as cricothyrotomy. In addition, administering 100% high-flow oxygen is imperative for patients presenting with cardiovascular or pulmonary symptoms to optimize tissue oxygenation (Sturm et al. 2018).

### Source removal and decontamination

Following airway stabilization, identifying and eliminating exposure to the inciting allergen is essential to halt progression. While gastrointestinal decontamination methods like gastric lavage are generally contraindicated due to inefficacy and the potential to delay other critical interventions, the rapid removal of external allergens remains a cornerstone of management (Gunasena & Jayawardana 2024).

### Hemodynamic support and fluid resuscitation

Early administration of intravenous fluids, in conjunction with the initial dose of adrenaline, is critical in patients experiencing cardiovascular compromise, as effective vasopressor activity may depend on adequate intravascular volume. Crystalloids are the preferred fluid choice, administered in boluses of 10 mL/kg (up to 500 mL) for children or 500 mL for adults, with repeated doses guided by clinical response. In severe cases with respiratory involvement, fluid resuscitation may be necessary after a second intramuscular dose of adrenaline (Muraro et al. 2022). Evidence, such as the findings from Ruiz-Garcia et al., highlights reversible cardiovascular dysfunction and associated gastrointestinal symptoms through early fluid administration. Intravenous fluids, therefore, play a vital role in stabilizing distributive shock and improving clinical outcomes (Ruiz-Garcia et al. 2021). In cases of anaphylaxis leading to distributive shock, fluid resuscitation is often effective. For adults, administer 1 to 2 L of isotonic saline, and for pediatric patients, deliver 10–20 mL/kg of isotonic fluids to address hypotension (Gunasena & Jayawardana 2024).

### Pharmacological approaches to treat anaphylaxis

#### Adrenaline

Epinephrine is the cornerstone and the first-line treatment option in anaphylaxis management, exerting potent effects on cardiovascular and respiratory stabilization via vasoconstriction, bronchodilation, and reduction of mucosal edema. Umasunthar et al. (2013) reported that initial treatment of anaphylaxis is stronger than the evidence base for the use of antihistamines and glucocorticoids in anaphylaxis (Dodd et al. 2021). It should be administered promptly following allergen removal and patient assessment, per established emergency protocols (Bilò et al. 2020). The initial adult dose is 0.3–0.5 mL of 1:1,000 concentration, while the pediatric dose is 0.01 mg/kg (up to 0.15 mg). Doses may be repeated every 5–10 min with refractory patients by intravenous infusion under continuous hemodynamic monitoring (Gunasena and Jayawardana 2024).

##### Route of administration

Intramuscular administration into the vastus lateralis is preferred for its superior bioavailability and rapid systemic absorption (Dreborg and Kim 2021) compared to subcutaneous or deltoid injection sites (Simons et al. 2001). Evidence indicates that intramuscular adrenaline achieves higher plasma concentrations compared to administration via a metered-dose inhaler (Breuer et al. 2013). In addition, while studies comparing intramuscular and subcutaneous adrenaline are influenced by injection site variability, they consistently demonstrate superior plasma adrenaline levels with the intramuscular route (Simons et al. 2001).

##### Special considerations and training

Intramuscular adrenaline remains the preferred route due to its safety profile and effectiveness; however, intravenous adrenaline may be necessary in exceptional scenarios such as refractory respiratory or cardiovascular compromise (Muraro et al. 2022). Healthcare professionals must be adequately trained in the administration of intramuscular adrenaline and its timely use, especially in patients with a history of life-threatening reactions. Furthermore, patients on beta-blockers may exhibit diminished responsiveness to adrenaline, underscoring the need for vigilant monitoring and individualized management strategies. For optimal outcomes, adrenaline should be supplemented with intravenous fluids in severe presentations and administered without delay. The clinical environment, such as emergency or critical care settings, plays a crucial role in enabling safe and effective use of intravenous adrenaline (Turner et al. 2021).

##### Efficacy and challenges of adrenaline in biphasic reaction

A 2020 systematic review by the European Academy of Allergy and Clinical Immunology (EAACI) assessed observational studies on adrenaline’s efficacy in critical outcomes, including mortality (De Silva et al. 2020). The findings underscored that timely and sufficient adrenaline administration leads to symptom resolution (Grabenhenrich et al. 2018), while delays are linked to prolonged reactions, hypotension, and fatalities. Although fatalities from anaphylaxis are rare (Nassiri et al. 2015), severe reactions are unpredictable, necessitating the treatment of all anaphylaxis episodes as potentially life-threatening. In the UK, approximately, one-third of the food-induced anaphylaxis deaths occur despite prompt adrenaline administration, often due to severe reactions requiring multiple doses (Patel et al. 2021). Around 10% of the anaphylaxis cases show inadequate response to a single dose, with most resolving after additional doses. Notably, anaphylaxis may recur hours later without re-exposure to allergens (biphasic reaction). A meta-analysis of 27 studies (2,758 patients) reported a 5% biphasic reaction rate, with no significant impact of adrenaline on reducing this risk. These findings highlight the need for vigilance and repeated interventions in severe cases (Lee et al. 2015, p. 45).

##### Early adrenaline administration: best practices and risks

Early administration of adrenaline is crucial upon anaphylactic signs and symptoms appear (ASCIA 2024). It is generally recommended to administer adrenaline as soon as anaphylaxis symptoms become evident, as delays exceeding 30 min are associated with a higher risk of biphasic reactions (Liu 2020). The intramuscular (IM) route is the preferred method for initial adrenaline administration (Panesar et al. 2013) due to its favorable safety profile, including for patients with cardiovascular comorbidities (Cardona et al. 2020). Conversely, intravenous (IV) adrenaline should be reserved for perioperative settings and administered only by experienced clinicians as an infusion rather than a bolus (Simons et al. 2014). A systematic review revealed that IV bolus administration increased the risks of adrenaline overdose and cardiovascular events compared to IM administration (Campbell et al. 2015). Excessive adrenaline doses, particularly via the IV route, can lead to severe complications such as tachyarrhythmias, hypertension, myocardial infarction, and stroke. However, in cases of imminent cardiac arrest, a bolus dose of IV or intraosseous adrenaline may be warranted (McLure et al. 2021).

##### Optimal adrenaline dosing

Crossover randomized controlled trials (RCTs) have demonstrated that 300 µg of IM adrenaline for children and 500 µg for adolescents and adults exhibit favorable absorption profiles (Simons et al. 2002). Adrenaline auto-injector recommendations include 0.15 mg for children weighing 7.5 kg, 0.3 mg for those weighing 25 to 30 kg, and at least 0.3 mg for adolescents (Patel et al. 2020) and adults for more favorable absorption profile (Duvauchelle et al. 2018). Limited data exist for children under 15 kg, though healthcare settings commonly administer a dose of 0.01 mg/kg (Muraro et al. 2007). Auto-injectors designed for children as small as 7.5 kg are licensed in some countries, including Germany. However, concerns exist regarding the risk of the needle striking the underlying bone in smaller children. Challenges such as limited availability, high costs, and supply shortages of auto-injectors remain prevalent in many regions (Kim et al. 2014).

##### Adrenaline auto-injectors

In terms of IM administration, the EAACI 2020 systematic review identified one study in which untrained caregivers were more able to give adrenaline correctly using a prefilled syringe than when using an adrenaline auto-injector (AAI) (Suwan et al. 2018), which reduces administration time by an average of 70 s compared to drawing up manually from an ampoule and results in fewer administration errors. Most AAIs deliver a maximum of 300 µg of epinephrine, although the recommended dose in teenagers and adults is 500 µg, resulting in significant underdosing that may lead to deadly results (Marie 2020). A prior RCT in food-allergic teenagers revealed that a 500 µg dosage of AAI had a more favorable pharmacokinetic and pharmacodynamic profile than 300 µg, without causing a higher rate of systemic adverse events (Patel et al. 2020). As a result, while certain settings may prefer to employ an AAI to provide an initial dose of adrenaline (for speed and convenience), subsequent doses should be administered via needle/syringe to ensure an appropriate dose (Dodd et al. 2021).

Adrenaline auto-injectors are recommended as the primary tool for managing anaphylaxis due to their advantages over traditional needle-syringe methods. These devices are user-friendly, relatively safe, minimize errors, and enable rapid administration. Their utilization in healthcare settings allows patients to familiarize themselves with the process, either by observing or practicing, enhancing their confidence in managing anaphylactic emergencies. They are designed for storage at 20–25℃, and auto-injectors have a limited shelf life due to adrenaline degradation, necessitating regular replacement (Muraro et al. 2022). In severe cases of anaphylaxis, clinical settings may warrant higher doses, such as 0.5 mg or repeated 0.3 mg doses, particularly for older adolescents or adults. This approach ensures effective treatment tailored to the severity of the reaction (Greenhawt et al. 2019).

##### Inhaled adrenaline

Inhaled adrenaline by a nebulizer concomitantly with oxygen is highly recommended when laryngeal/pharyngeal edema is suspected. But due to the negligible systemic absorption of inhaled adrenaline, IM adrenaline shot should be taken (Hershko et al. 2023). Schlegel et al. (2009) evaluated the utility of including epinephrine inhalers in emergency kits for patients with anaphylaxis and concluded that there was insufficient evidence to add aerosolized epinephrine to emergency kits due to poor delivery to the lungs and low absorption, with the majority of inhaled epinephrine ending up in the oropharynx and being broken down by the GI tract. Furthermore, most children complained about the flavor and reported coughing and dizziness. The findings of this study imply that the potential advantage of inhaled epinephrine (Simons et al. 2000).

##### Intranasal adrenaline

Intranasal (IN) administration of adrenaline presents a promising alternative to intramuscular (IM) delivery, leveraging the highly vascularized nasal mucosa for rapid systemic absorption (Srisawat et al. 2022). This route may offer significant advantages in terms of patient acceptance, minimizing the psychological distress associated with needle-based administration. In addition, the nasal route is associated with minimal adverse effects and has limited contraindications, such as facial trauma and epistaxis (Bailey et al. 2017). Animal studies have demonstrated the systemic absorption of IN epinephrine with higher plasma concentrations observed for a 5 mg IN dose compared to a 0.3 mg IM dose within the first minute post-administration. These findings highlight its potential for faster onset with reduced risks of tachycardia (Dretchen et al. 2020). Furthermore, human trials have corroborated the efficacy of IN epinephrine in achieving plasma concentrations comparable to IM administration, without significant or sustained adverse effects. However, concerns remain regarding the impact of anaphylaxis-related histamine release, which can induce nasal mucosal edema, potentially impairing the absorption of IN epinephrine. Despite this limitation, the convenience and efficacy of IN administration make it a viable alternative, particularly in scenarios where IM delivery is not feasible or practical (Tuttle et al. 2020).

##### Sublingual adrenaline

Another conceivable route being studied is sublingual delivery of epinephrine, which must be absorbed by the buccal mucosa in order to enter the bloodstream. Compared to injectable epinephrine, a quickly disintegrating sublingual pill would have a longer shelf life of up to 7 years and enable smaller dosages (Rawas-Qalaji et al. 2013). It was hypothesized that instead of administering a 0.15 mg intramuscular dosage of epinephrine to treat pediatric anaphylaxis, they might be able to utilize a 30 mg sublingual tablet that dissolves quickly. Since oral and sublingual mucosal swelling in anaphylaxis may impact the rate and extent of epinephrine absorption, it will be crucial to observe how the sublingual route compares to intramuscular (IM) injection in human clinical trials and during anaphylactic episodes (Rachid 2013).

##### Novel approaches to the traditional IM device

Other studies have attempted to solve problems with epinephrine intramuscular delivery, such as patient convenience, mobility, and shelf life with innovative devices. For instance, ZENEO® is a needleless intramuscular device and the most recent experimental substitute for IM auto-injectors. The auto-injector is a prefilled single-use needle-free device currently under development (Alvarez-Perea et al. 2023). The drug is delivered intramuscularly via the device, which propels it at high enough pressure and speed to pass through skin. This portable, needle-free device might improve the outcome by mitigating needle anxiety in patients and parents and possibly making training easier (Sonoda et al. 2023).

#### Corticosteroids

Their precise effectiveness in anaphylaxis remains uncertain, and they show therapeutic benefit in conditions such as reactive airway diseases. For acute management, intravenous methylprednisolone (80–125 mg) or hydrocortisone (250–500 mg) is administered. Following the acute phase, oral prednisone (40–60 mg daily or divided into two doses) is recommended for 3–5 days. In situations involving unidentified allergens or delayed follow-up, a tapering regimen over 2 weeks may be appropriate. Methylprednisolone and dexamethasone are chosen since they have minimal mineralocorticoids like action abrogating the risk of fluid retention. Corticosteroids are recommended as a third-line treatment for underlying asthma or shock since their major action is to reduce the late-phase inflammatory response (Karunarathna et al. 2024). Given corticosteroids’ sluggish absorption kinetics and mode of action, it is theoretically improbable that they are useful in the acute treatment of anaphylaxis. In contrast, following systematic reviews have reaffirmed the paucity of evidence that corticosteroids lower reaction severity or prevent biphasic reactions (Alqurashi & Ellis 2017).

Corticosteroids are more frequently administered than adrenaline in the acute management of anaphylaxis which may inadvertently delay or distract from the timely administration of adrenaline (Dubus et al. 2019). Corticosteroids may offer therapeutic value in specific scenarios, such as refractory anaphylaxis and anaphylaxis occurring in the setting of poorly controlled asthma. Therefore, incorporating corticosteroids as an adjunctive treatment for refractory anaphylaxis is reasonable, but they should not replace adrenaline or other inotropic or vasopressor agents in the treatment hierarchy (Dodd et al. 2021).

Recent Cochrane reviews have assessed the efficacy and safety of glucocorticoids in acute anaphylaxis management, concluding that there is insufficient evidence to support their routine use in the emergency department or for the prevention of biphasic reactions (Choi et al. 2019). High-dose glucocorticoids (500–1,000 mg in adults) may be considered for patients presenting with generalized urticaria, coexisting asthma, airway edema, or stridor after stabilization (Böhm et al. 2018; Kim et al. 2014). In pediatric patients, prednisolone at a dose of 2 mg/kg can be administered via suppositories, enemas, or injectable formulations as appropriate (Quoc et al. 2021). Table 2 recapitulates the dosage and administration of three common parenteral glucocorticoids: hydrocortisone, methylprednisolone, and dexamethasone.

**Table 2:** Dosage and administration of corticosteroid injections (Amir Rawa et al. 2022)

Agent	Dose	Adults min. dose	Children max. dose	Notes
Hydrocortisone IV or IM	2–4 mg/kg	200 mg	100 mg	Rapid onset, but may contain alcohol, which may be hazardous in the management of anaphylaxis
Methylprednisolone IV or IM	1–2 mg/kg	100 mg	50 mg	Rapid onset, but may not be available at the primary care level
Dexamethasone IV or IM	0.1–0.4 mg/kg	20	10	Slow onset, inexpensive, widely available

#### Antihistamines

Antihistamines, especially H1 receptor blockers, are commonly utilized to manage allergic reactions, including anaphylaxis. Diphenhydramine, the most widely used H1 blocker, is typically administered at 25–50 mg intravenously or intramuscularly. Besides its role in anaphylaxis, management remains uncertain, and it is effective in addressing milder allergic symptoms. In more severe reactions, combining H1 blockers with H2 receptor antagonists such as ranitidine (50 mg IV over 5 min) or cimetidine (300 mg IV) may provide additional benefit, as histamine affects multiple receptor subtypes. However, cimetidine requires careful use in patients with renal or hepatic dysfunction or those on beta-blockers due to potential drug interactions and side effects. Once the patient is stabilized, transitioning from intravenous to oral antihistamines is advisable if ongoing treatment is needed (Gunasena & Jayawardana 2024). Oral H1-antihistamines, such as dimetindene and clemastine, which are also available in IV formulations, can alleviate cutaneous symptoms of anaphylaxis and rhinoconjunctivitis (Simons et al. 2015).

##### Antihistamines limitations in allergic reactions including anaphylaxis

The ASCIA 2024 Guidelines reported that antihistamines should not be used as part of the primary emergency treatment for anaphylaxis as they have no role in treating respiratory or cardiovascular symptoms of anaphylaxis (ASCIA 2024). Their primary utility lies in managing cutaneous symptoms often associated with allergic reactions, including anaphylaxis. However, there is no robust evidence from randomized controlled trials (RCTs) to support their use in anaphylaxis, and they do not prevent biphasic reactions (Simons et al. 2015). In addition, H1-antihistamines, particularly when administered rapidly via intravenous bolus, may cause sedation, potentially masking anaphylaxis symptoms, and can precipitate hypotension (Nurmatov et al. 2014). Other reports recommend relegating antihistamines to second- or third-line intervention as a concern for delaying the administration of both initial and subsequent doses of adrenaline (Cardona et al. 2020).

Data from studies, including the Cross-Canada Anaphylaxis Registry (C-CARE), suggest that prehospital antihistamine use may be associated with lower administration rates of multiple adrenaline doses and delayed presentation to healthcare facilities, potentially increasing morbidity. In a large national prospective study, the Cross-Canada Anaphylaxis Registry (C-CARE) analyzed 3,498 cases of anaphylaxis over 6 years. Prehospital use of antihistamines was linked to hospitalization, intensive care admission, and intravenous fluid requirements (Gabrielli et al. 2019). Antihistamines are also ineffective in preventing biphasic reactions (Shaker et al. 2020). Furthermore, data from the European Anaphylaxis Register, which analyzed 9,171 anaphylaxis episodes, revealed a significant link between antihistamine use and a higher incidence of biphasic reactions (Kraft et al. 2020), likely due to the delay in administering adrenaline. Consequently, antihistamines are not recommended for the acute management of anaphylaxis (ASCIA 2024).

##### Antihistamines: supportive, not primary, in anaphylaxis management

The ASCIA 2020 guideline reported that antihistamines may be useful for persistent skin symptoms after anaphylaxis has been resolved but should only be administered once the acute reaction has been effectively treated with adrenaline and other first-line interventions (Cardona et al. 2020)). A recent meta-analysis suggested that antihistamines and glucocorticoids may help prevent reactions related to chemotherapy but not those triggered by radiocontrast media (Shaker et al. 2020). In addition, antihistamines are effective in reducing reactions during allergen immunotherapy (Roberts et al. 2018).

#### Misplaced priorities: overuse of corticosteroids and antihistamines

Research indicates that many physicians prioritize corticosteroids and antihistamines as first-line treatments for anaphylaxis, with this practice being even more prevalent among specialists. Studies reveal that these medications are administered more frequently than epinephrine, despite guidelines emphasizing epinephrine as the primary treatment. For instance, in a study involving 1,970 children with anaphylaxis, antihistamines and corticosteroids were used in 76% and 82% of cases, respectively, while epinephrine was utilized in only 28% (Grabenhenrich et al. 2016). Similarly, other studies reported antihistamine usage rates between 81.5% and 93% and corticosteroid administration in 55.5% to 73% of cases, compared to lower epinephrine use (46%–58.3%) (Sidhu et al. 2016). Although corticosteroids and antihistamines play a supportive role in managing refractory symptoms or preventing biphasic reactions, they are adjunctive treatments rather than first-line options (Muraro et al. 2014).

#### Bronchodilators

Bronchodilators play a vital supportive role in managing anaphylaxis accompanied by bronchospasm, especially in individuals with underlying respiratory conditions like asthma. Inhaled beta-agonists, such as albuterol or a combination of ipratropium bromide and albuterol, are the drugs of choice for wheezing (Gunasena and Jayawardana 2024) but caution that they are ineffective in addressing upper airway obstruction, hypotension, or shock. As a result, their role is strictly as adjunctive therapy rather than a primary treatment option (Cardona et al. 2020). For persistent bronchospasm unresponsive to standard therapy, intravenous magnesium sulfate may be used, adhering to dosing protocols similar to those for severe asthma exacerbations (Gunasena and Jayawardana 2024). In addition, Beta-2 agonists like salbutamol may serve as adjunctive therapy for addressing lower respiratory symptoms and significant bronchial obstruction during anaphylaxis. These should be administered following initial intramuscular adrenaline treatment, ideally using an oxygen-driven nebulizer or a metered-dose inhaler with a spacer for optimal delivery (Muraro et al. 2022). In cases of ongoing respiratory symptoms during anaphylaxis, beta-2 agonists (administered via inhalation or parenterally) should not replace additional parenteral adrenaline treatment. According to guidelines from RCUK (2008), WAO (2011/2020), EAACI (2014), and ASCIA (2024), bronchodilators are emphasized as supplementary therapies to support adrenaline, rather than as substitutes. But in case of mild or moderate respiratory symptoms, beta-2 agonists can be delivered using a metered-dose inhaler (MDI) with a large-volume spacer with no need for oxygen. However, there is limited evidence supporting the use of MDIs with spacers in cases of acute severe or life-threatening respiratory symptoms. In such situations, beta-2 agonists should be administered using an oxygen-driven nebulizer for optimal effectiveness (Payus et al. 2018).

International guidelines agreed that bronchodilators may be helpful for persisting wheeze, but caution that they do not prevent or relieve upper airway obstruction, hypotension or shock, and should, therefore, be used as adjunct treatments. Inhaled beta-2 agonists are commonly utilized as second-line treatments for anaphylaxis, although evidence supporting their effectiveness in this context is limited and largely derived from their use in acute asthma management (Simons et al. 2011). There are anecdotal reports of anaphylaxis first misdiagnosed as severe asthma, which did not respond to parenteral β-2 agonists therapy but did respond to adrenaline (Payus et al. 2018). For this reason, IV salbutamol must not be used in preference to adrenaline for acute anaphylaxis (Dodd et al. 2021).

#### Vasopressors and glucagon

Vasopressors may be considered for patients needing multiple doses of epinephrine but who develop significant side effects, such as arrhythmias or chest pain, during intravenous epinephrine infusion. Although no specific vasopressor is universally endorsed as a second-line option for anaphylaxis, their use typically aligns with established protocols for treating other types of hypotensive shock (Karunarathna et al. 2024). Parenteral glucagon can provide supportive benefits for patients who do not respond adequately to epinephrine or for those on β-blockers (Parish et al. 2019).

#### Omalizumab

Omalizumab, an anti-IgE monoclonal antibody, reduces the expression of high-affinity IgE receptors (FcεRI) on basophils, mast cells, and dendritic cells, thereby inhibiting the IgE-mediated signaling cascade (Benito-Villalvilla et al. 2023). It has been widely used in the management of moderate to severe asthma and chronic spontaneous or inducible urticaria (Kabashima et al. 2018). More recently, its applications have expanded to include severe food allergies, eosinophilic gastroenteritis, acute reactions during rush immunotherapy, mast cell disorders, and idiopathic anaphylaxis (Imakiire et al. 2020).

Anaphylaxis management involves both acute interventions to alleviate symptoms and long-term strategies to prevent recurrence. Long-term approaches include avoiding known triggers through specific immunotherapy or modulating the IgE response. In cases of food-related anaphylaxis, anti-IgE therapy has emerged as a promising option. Numerous case series have demonstrated its clinical efficacy when used either as a standalone treatment or in combination with oral immunotherapy (Quoc et al. 2021).

#### Allergen immunotherapy and drug desensitization

Immunomodulatory approaches should be considered in venom immunotherapy and drug desensitization (Kim et al. 2014). Indeed, patients with VIA show the best response to subcutaneous venom immunotherapy for preventing anaphylaxis in both children and adults (Monsieurs et al. 2015). For drug desensitization, administration of drugs (with increasing doses) can achieve a tolerant state to targeted drug doses. Desensitization can be successful for patients with anaphylaxis induced by chemotherapy, biologics, NSAIDs, and antibiotics (Maris et al. 2021).

#### Investigational therapeutic agent: sirtuin 6

Sirtuin 6 (SIRT6), a NAD-dependent deacetylase, is being explored as a potential therapeutic target for anaphylaxis. Its mechanism involves inhibiting the transcription of protein tyrosine phosphatase receptor type C, which in turn downregulates the FcERI signaling pathway in mast cells. By suppressing mast cell activation, SIRT6 activation could help mitigate the severity of anaphylactic reactions, offering a promising new approach under investigation for anaphylaxis treatment (Karunarathna et al. 2024).

## Emergency response to anaphylaxis

**Fig. 2:**
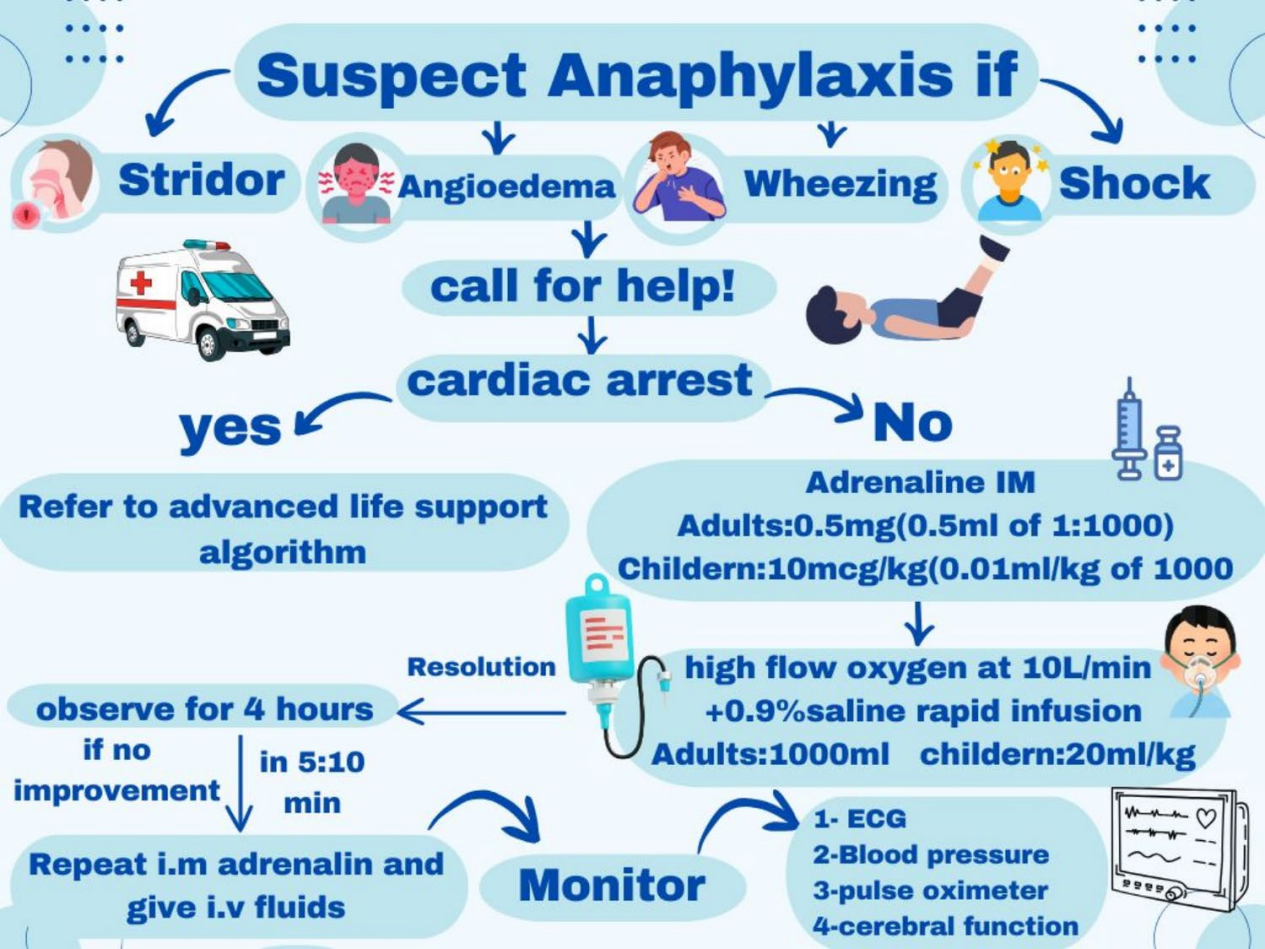
Systematic representation for preliminary management of anaphylaxis

When anaphylaxis is suspected, prompt actions is crucial as shown in Fig. 2. The patient’s airway, breathing, circulation, and mental status should be assessed immediately. If the allergen is identifiable, efforts should be made to remove it. Assistance should be sought from nearby individuals or caregivers, and an emergency call should be placed without delay. Alternatively, the patient should be taken directly to an emergency department for medical care (Amir Rawa et al. 2022). While awaiting the arrival of emergency responders, any suspected allergen should be removed if feasible.

In cases of anaphylaxis, individuals should be positioned in supine position to enhance venous return to the heart or seated upright if experiencing respiratory distress. If vomiting occurs, the head should be turned slightly downward, and any material obstructing the airway should be promptly cleared to prevent aspiration. Epinephrine auto-injector should be used if available according to the instructions provided on the packaging (Alsabri et al. 2024). Infants should not be held upright; however, if breathing is compromised, the patient may be allowed to sit to ease respiratory effort (Alsabri et al. 2024). For pregnant patients, positioning on the left side with the bed tilted in a head-down position is recommended to optimize venous return and reduce pressure on the inferior vena cava. Sudden changes to an upright posture should be avoided (Tan et al. 2022).

Epinephrine should be administered immediately after removing the trigger, as it plays a critical role in stabilizing cardiovascular and respiratory functions by enhancing vasoconstriction, peripheral vascular resistance, bronchodilation, and reducing mucosal edema (Böhm et al. 2018). Following epinephrine administration, the patient should be evaluated in accordance with established emergency protocols (Cardona et al. 2020). Timely administration of adrenaline has been shown to decrease the likelihood of biphasic reactions (Liu et al. 2020). Upon hospital arrival, continuous monitoring of vital signs including electrocardiogram (ECG), pulse, and blood pressure is essential to assess the severity of the reaction, detect signs of shock, and evaluate the risk of biphasic reactions (Monsieurs et al. 2015). Patients exhibiting cardiovascular or respiratory involvement should receive high-flow oxygen (100%) to support critical functions (Takazawa et al. 2021). In cases of snake envenomation, administration of antivenom should be promptly initiated as per clinical guidelines (Muraro et al. 2022).

## Supportive measures for anaphylaxis

Supportive care is integral to the management of anaphylaxis and achieving favorable patient outcomes. This involves continuous monitoring of vital parameters, including pulse, blood pressure, electrocardiogram (ECG), and oxygen saturation. High-flow oxygen supplementation should be provided to maintain oxygen saturation between 94 and 98% (Arroabarren et al. 2011). In cases of hypotension or inadequate response to initial therapy, intravenous (IV) access should be established, and a rapid fluid bolus of 10 mL/kg administered (Simons 2009). For the symptomatic management of vomiting, diphenhydramine may be given intramuscularly or intravenously at a dose of 1 mg/kg, not exceeding 50 mg per dose (Arroabarren et al. 2011). In addition, ranitidine can be administered orally or intravenously at the same dose (1 mg/kg), with a maximum of 50 mg per dose (Clark et al. 2023).

In cases of respiratory failure or significant respiratory distress caused by airway edema or bronchospasm, advanced airway management should be initiated promptly, either through endotracheal intubation or the insertion of a supraglottic airway device. A tracheotomy may be warranted if intubation proves unsuccessful (Amir Rawa et al. 2022). Concurrently, IM epinephrine should be administered promptly in cases of GRADE II anaphylaxis or higher, using the recommended dosages (Amir Rawa et al. 2022; Kim et al. 2018). If there is no clinical improvement, the dose may be repeated every 5–15 min as needed. For patients classified as GRADE IV, experiencing or at imminent risk of cardio-respiratory arrest, IV bolus epinephrine is indicated (Hammad et al. 2022).

For GRADE II and GRADE III anaphylaxis patients with established venous access and under close monitoring, such as in an ICU or perioperative setting, IV bolus epinephrine may be considered as outlined in Table 3. If there is no clinical improvement within 3–5 min for GRADE IV patients or 1–2 min for GRADE II and III patients, an additional dose of IV bolus epinephrine should be administered (Pouessel et al. 2024).

**Table 3:** Recommended doses of IV bolus of epinephrine in anaphylaxis

Grade	Dose recommended
GRADE II	0.01–0.05 mg for patients ≥ 14 years old; 0.001–0.002 mg/kg (1–2 μg/kg) for patients < 14 years old
GRADE III	0.1–0.2 mg for patients ≥ 14 years old; 0.002–0.01 mg/kg (2–10 μg/kg) for patients < 14 years old
GRADE IV	1 mg for patients ≥ 14 years old; 0.01–0.02 mg/kg for patients < 14 years old

For those with GRADE IV anaphylaxis, IV epinephrine infusion can commence once stabilization begins, even if cardiopulmonary symptoms persist incompletely resolved. The dose of epinephrine IV infusion should be 3–30 μg/kg/h. During IV epinephrine administration, continuous monitoring of electrocardiogram (ECG), blood pressure (BP), respiratory rate, and oxygen saturation is essential. In cases where bronchospasm persists or stridor develops following epinephrine administration, high-dose nebulized budesonide may be considered as an adjunct treatment. For patients exhibiting circulatory compromise, fluid resuscitation is advised, starting with an initial bolus of 20 mL/kg, with subsequent adjustments based on clinical response. Following appropriate intervention, those patients should undergo hospital monitoring for a minimum of 12 h. Key parameters to observe include heart rate, blood pressure, respiratory rate, oxygen saturation, and urine output (Amir Rawa et al. 2022).

## Natural products with anti-anaphylaxis potential

### German chamomile extract

The methanolic extract of *Matricaria recutita* L., German chamomile was found to effectively inhibit mast cell histamine release in a mast cell mediated allergy model in vitro, although more research is required to define its mechanism of action (Chandrashekhar et al. 2011). The pharmacokinetic parameters of quercetin, luteolin, and apigenin after oral administration of *M. chamomilla* extract in rats were determined, where the results showed that all three compounds were rapidly absorbed, with T_max_ values of 0.79 h, 0.42 h, and 0.51 h for quercetin, luteolin, and apigenin, respectively. Luteolin exhibited the highest systemic exposure (C_max_ = 3.04 μg/ml, AUC_0–∞_ = 19.89 μg*h/ml), followed by apigenin (C_max_ = 0.42 μg/ml, AUC_0–∞_ = 5.03 μg*h/ml) and quercetin (C_max_ = 0.29 μg/ml, AUC_0–∞_ = 3.88 μg*h/ml). Despite their rapid absorption, elimination was relatively slow, with half-lives (T_1/2_) of 13.60 h for quercetin, 4.43 h for luteolin, and 8.82 h for apigenin, suggesting prolonged systemic retention (Dong et al. 2017**)**. However, till the time of this review, the pharmacokinetics of chamomile in humans is not well described (Kimura et al. 2024**)**, requiring further studies in humans to establish clinical relevance.

### Impatiens textori extract

The 35% ethanol extract of the flowers of *Impatiens textori MIQ.* exhibited anti-anaphylactic and anti-pruritic effects. Its key bioactive compounds, including apigenin, apigenin 7-glucoside, and luteolin, were shown to significantly inhibit blood pressure decreases in both IgE-dependent and IgE-independent anaphylaxis models. The extract of the flowers of *I. textori* also mitigated the reduction in blood flow caused by antigen-induced anaphylaxis in sensitized mice. Beyond anaphylaxis, the extract effectively reduced scratching behavior induced by platelet-activating factor (PAF), serotonin (5-HT), and proteases without central depressant effects (Ueda et al. 2005). However, to date, a literature search for pharmacokinetic studies on *I. textori* yielded no relevant publications in animal or human models. Thus, future research should focus on elucidating the pharmacokinetics of *I. textori* to support its safe and effective use in medicinal applications.

### Kincung flower extract

The ethanol extract of *Etlingera elatior*—Kincung flower—was found to suppress allergic reactions by inhibiting mast cell degranulation and lowering serum IL-4 and IgE levels in sensitized mice. Its potential effectiveness in controlling type I hypersensitivity and active cutaneous anaphylaxis is also shown by its reduction of basophil and eosinophil infiltration at inflammatory sites (Elidahanum et al. 2020). However, further studies on the pharmacokinetics, bioavailability, and molecular mechanism of *E. elatior* are needed (Juwita et al. 2018).

### Litsea cubeba essential oil

*Litsea cubeba*, also known as Makauy, is a traditional herb with diverse therapeutic uses, including anti-anaphylaxis and anti-inflammatory effects. The essential oil of *L. cubeba* (LCEO), rich in citral (neral and geranial), demonstrated immunosuppressive effects by reducing TNF-α and IL-12 production in dendritic cells (DCs). LCEO also inhibited contact hypersensitivity (CHS) responses and decreased T-cell infiltration in inflammatory sites (Chen et al. 2016; Qian et al. 1980).

### Patchouli oil from *Pogostemon cablin*

Patchouli oil, the essential oil of *Pogostemon cablin* (Blanco) Benth. (Lamiaceae), and its main component, patchouli alcohol, were found to show anti-allergic action by lowering mast cell degranulation, reducing cytokine generation, and thus modifying dendritic cell responses. Supported by delay in hypersensitivity models and inhibition of passive cutaneous anaphylaxis, their therapeutic effects reach to reducing systemic inflammation and hypersensitivity (Chen et al. 2013; He et al. 2013; Su et al. 2015). The pharmacokinetics of patchouli alcohol, a tricyclic sesquiterpene and a critical bioactive ingredient of patchouli oil, have been extensively studied in several reports, demonstrating a two-compartment distribution with linear elimination kinetics. However, oral bioavailability remains limited due to poor solubility and first-pass metabolism (Hu et al. 2017; Zhang et al. 2016; Zhu et al. 2024a, b).

### Clinacanthus nutans extract

*Clinacanthus nutans* (Burm. f.) Lindau water leaf extract was found to downregulate lipid metabolism and improve propanoate and amino acid pathways in an in-vivo ovalbumin-induced active systemic anaphylaxis model, displaying anti-anaphylactic effects. Supported by ^1^H-NMR metabolomics approach, these results draw attention to the extract’s overall systemic regulating action on allergic mechanisms (Elgendy 2017). The in-vitro effect of *C. nutans* leaves methanol extract on the activity of CYP3A4 and CYP2E1 in human liver microsomes was evaluated, showing significant inhibition (Quah et al. 2017).

### Isoquercitrin and hyperin flavonoids from persimmon peel

Isoquercitrin and hyperin, isolated from the peel of persimmon, showed anti-anaphylactic action by inhibiting mast cell degranulation, mainly by lowering the intracellular calcium (Ca^2^⁺) elevation, which is a necessary trigger for histamine release. Their mechanism is direct scavenging of reactive oxygen species (ROS) generated by NADPH oxidase (NOX), so reducing the oxidative stress related with allergic reactions (Itoh et al. 2011). Isoquercetin was also found to have a significant inhibitory effect on histamine release from rat mast cells (Park 2020). These results place isoquercitrin and hyperin as potential candidates for reducing type I allergic reactions by means of their combined antioxidant and anti-inflammatory action. The bioavailability of isoquercitrin was found to be better than quercetin by 2.35 times, and first concentration peak of quercetin conjugates appear 60–90 min after the intake of isoquercitrin, followed by the second, higher peak at 2.5–4 h due to enterohepatic circulation (Valentová et al. 2014).

### Spinacetin flavonoid from *Inula japonica*

Derived from *Inula japonica* Thunb., spinacetin reduced mast cell activation and passive cutaneous anaphylaxis (PCA) in mice. Spinacetin also reduced the synthesis of inflammatory mediators including leukotriene C4 (LTC4) and interleukin-6 (IL-6) as well as histamine release. Spinacetin was found to interact with signaling pathways including Syk, LAT, PLCγ, MAPKs, and NF-κB. Spinacetin’s oral administration dose-dependently attenuated IgE/Ag-mediated PCA reaction in mouse model (Ji et al. 2018). The computational pharmacokinetic profile of spinacetin showed it can be well absorbed by oral intake, without passing the blood brain barrier or becoming a P-glycoprotein substrate (Jose et al. 2022).

### Naringenin

Naringenin, a citrus-derived flavonoid, exhibited anti-anaphylactic activity by targeting immune and inflammatory pathways. Studies have shown that naringenin suppresses the activation and migration of macrophages, key players in allergic inflammation. In addition, it inhibits the production of pro-inflammatory mediators such as MCP-1, NO, and TNF-α in LPS-stimulated RAW 264.7 macrophages (Fang et al. 2010; Hirai et al. 2007). Naringenin exhibited dose-proportional pharmacokinetics in humans, and T_max_ occurring at 2.41–3.17 h, while the half-life (t_1/2_) ranged from 2.65 to 3.0 h, indicating rapid clearance. No adverse effects were observed with a suggested 300 mg twice daily dosing (Rebello et al. 2020).

### Luteolin

Flavonoids are widely known for their anti-inflammatory and anti-anaphylactic properties, including luteolin and quercetin derivatives (Middleton et al. 2000). Luteolin, from *Folium perillae* and *Flos lonicerae*, was found to reduce phosphodiesterase 4 (PDE-4), thus lowering cyclic AMP (cAMP) hydrolysis and preventing neutrophil adhesion and cytokine release (Jiang et al. 2015). Luteolin is rapidly absorbed following oral intake in rats through the intestine in its aglycone form following hydrolysis of luteolin glucoside by the sodium glucose co-transporter 1 (SGLT1). However, further research is necessary to fully characterize the pharmacokinetic profile of luteolin in humans (Wang et al. 2021a, b).

### Wogonin

Wogonin, a flavonoid isolated from *Radix scutellariae*, exhibits significant anti-anaphylactic effects alongside a broad range of pharmacological activities, including anti-inflammatory and antiviral properties. Screening using a two-dimensional online cell membrane chromatography method revealed that wogonin had specific activity on the epidermal growth factor receptor (EGFR). In-vitro studies demonstrated its dose-dependent inhibition of high EGFR-expressing cell viability, suggesting immunomodulatory properties (Shalaby et al. 2023). However, wogonin displays very low oral bioavailability (1.10%), likely resulting from poor solubility and extensive first-pass metabolism (Hassanin et al. 2019).

### Rosmarinic acid

Found in many herbs, rosmarinic acid was found to lower inflammatory markers and to exert anti-anaphylaxis activity in atopic dermatitis, asthma, allergic rhinitis, and ovalbumin (OVA)-induced intestinal allergies. It was found to significantly lower IgE, histamine, and mast cell proteinase levels in serum of OVA challenged mice while raising antioxidant enzyme activity. The ability of rosmarinic acid to balance pro- and anti-inflammatory cytokines emphasizes its therapeutic value in control of food allergies and anaphylaxis (Jia et al. 2023). Upon oral intake, rosmarinic acid is mainly metabolized by the gut microflora into simple phenolic units. Its elimination is mainly through renal excretion, with no serious adverse effects of herbal remedies containing rosmarinic acid reported (Hitl et al. 2020).

### Isoxazolines from *Xanthoceras sorbifolia*

Two isoxazolines isolated from *Xanthoceras sorbifolia* Bunge fruit husks, 1-oxa-2-azaspiro[4.5]dec-2-ene-8*β*-ol and 1-oxa-2-azaspiro[4.5]dec-2-ene-8*α*-ol, showed notable in-silico anti-anaphylaxis potential by phosphodiesterase IV (PDE-4) inhibition, modulating inflammatory pathways involved in anaphylaxis. The binding affinity and stability of these molecules revealed by computational modeling and molecular docking studies exceeded the clinical PDE-4 inhibitor Rolipram (Ge et al. 2016).

### Lipoic acid

Lipoic acid was evaluated for its effects on soybean β-conglycinin-induced hypersensitivity in a rat model. Administration of 25 mg/kg lipoic acid significantly reduced β-conglycinin-specific IgE and histamine levels in serum and the jejunum. In addition, lipoic acid supplementation did not affect interferon-γ or interleukin-4 levels, indicating its effects might be independent of typical Th1/Th2 cytokine modulation. These findings highlight its potential as an immunomodulator for managing food-induced hypersensitivity reactions, such as soybean allergies (Han et al. 2010).

### Fungal immunomodulatory protein from *Lignosus rhinocerotis*

A novel fungal immunomodulatory protein (FIP-Lrh) from *Lignosus rhinocerotis*, commonly known as Tiger Milk mushroom, exhibited promising anti-anaphylactic and immunomodulatory properties. FIP-Lrh was found to strongly bind to glycoproteins such as N-acetylgalactosamine and N-acetylglucosamine, which are crucial for immune cell interactions. Functional studies have confirmed its potential to modulate immune responses, making it a candidate for both preventive and therapeutic applications in managing anaphylaxis and other immune-related disorders (Pushparajah et al. 2016).

### Phenanthrenes and stilbenes from* Gymnadenia conopsea* tubers

The methanolic extracts of the tubers of *Gymnadenia conopsea* containing stilbenes and phenanthrenes were found to inhibit antigen-induced degranulation by 65.5 to 99.4% at 100 μM in RBL-2H3 cells. Furthermore, the methanolic extract significantly inhibited allergic reactions of ear passive cutaneous anaphylaxis in mice (Matsuda et al. 2004).

### Picroliv ididoid glycosides from *Picrorhiza kurroa*

Picroliv, a standardized iridoid glycoside fraction derived from the root and rhizomes of *Picrorhiza kurroa,* inhibited passive cutaneous anaphylaxis (PCA) in mice and rats at 25 mg/kg. Though it was not found to directly act as a histamine receptor antagonist, it lowered mast cell degranulation and prevented Schultz–Dale reactions in sensitized guinea pig ileum preparations (Baruah et al. 1998).

### Alginic acid

Made from seaweed, alginic acid showed strong anti-anaphylactic action by blocking histamine release and nuclear factor-κB (NF-κB) activation suppression. In mice, experimental studies found that it significantly lowered both systemic and passive cutaneous anaphylaxis. It also reduced the expression of inflammatory cytokines TNF-α and IL-1β in mast cells, so positioning it as a potential natural treatment for anaphylaxis and associated inflammatory diseases (Jeong et al. 2006). Table 4 categorizes the discussed natural products by their chemical nature and summarizes their mechanisms of action and experimental evidence.

**Table 4:** Summary of natural products with anti-anaphylaxis potential

Extracts			References
Natural product	Source	Experimental findings	
German chamomile extract	*Matricaria recutita*	Inhibition of mast cell degranulation Suppressed histamine release in vitro	(Chandrashekhar et al. 2011)
*Impatiens textori* extract	*Impatiens textori MIQ*	Stabilization of mast cells, inhibition of PAF and serotonin effects Reduced BP decrease and scratching behavior in mice	(Ueda et al. 2005)
Kincung flower extract	*Etlingera elatior*	IL-4 and IgE suppression, reduction in eosinophil and basophil infiltration Lowered serum IL-4 and IgE in sensitized mice	(Elidahanum et al. 2020)
*Litsea cubeba* essential oil	*Litsea cubeba*	TNF-α and IL-12 suppression, inhibition of T-cell infiltration Reduced CHS responses and dendritic cell activity in mice	(Chen et al. 2016; Qian et al. 1980)
*Clinacanthus nutans* extract	*Clinacanthus nutans*	Downregulation of lipid metabolism, improvement of amino acid pathways Alleviated OVA-induced anaphylaxis	(Elgendy 2017)

Phenolics			
Natural product	Source	Experimental findings	Reference
Isoquercitrin, hyperin	Persimmon peel	ROS scavenging, inhibition of Ca^2^⁺ elevation, mast cell degranulation Reduced histamine release in rat mast cells	(Itoh et al. 2011; J. Park 2020)
Spinacetin	*Inula japonica*	Inhibition of Syk, LAT, PLCγ, MAPKs, NF-κB, and histamine release Reduced IgE-mediated PCA in mice	(Ji et al. 2018)
Naringenin	Citrus fruits	Suppression of macrophage activation, cytokine inhibition (MCP-1, NO, TNF-α) Reduced inflammation in LPS-stimulated macrophages	(Fang et al. 2010; Hirai et al. 2007)
Luteolin	*Folium perillae*, *Flos lonicerae*	Inhibition of PDE-4, suppression of cAMP hydrolysis Prevented neutrophil adhesion and cytokine release	(Jiang et al. 2015; Middleton et al. 2000)
Rosmarinic acid	Various herbs	Balancing pro-/anti-inflammatory cytokines, lowering IgE and histamine Reduced inflammatory markers in OVA-induced allergic mice	(Jia et al. 2023)

Alkaloids			Reference
Natural product	Source	Experimental Findings	
Isoxazolines	*Xanthoceras sorbifolia*	PDE-4 inhibition, modulation of inflammatory pathways Computational modeling showed higher binding affinity than Rolipram	(Ge et al. 2016)
Other natural products			
Natural product	Source	Experimental findings	Referrences
Patchouli oil	*Pogostemon cablin*	Mast cell degranulation suppression, cytokine inhibition Reduced PCA and hypersensitivity in animal models	(Chen et al. 2013; He et al. 2013; Su et al. 2016)
Lipoic acid	Organosulfur compound	IgE and histamine suppression, villus height increase Reduced β-conglycinin-specific IgE and histamine levels	(Han et al. 2010)
FIP-Lrh protein	*Lignosus rhinocerotis*	Glycoprotein binding, immune modulation Functional recombinant protein produced in *E. coli* cells	(Pushparajah et al. 2016)
Phenanthrenes, stilbenes	*Gymnadenia conopsea*	Inhibition of antigen-induced degranulation Reduced PCA in RBL-2H3 cells and mouse ear models	(Matsuda et al. 2004)
Picroliv	*Picrorhiza kurroa*	Mast cell stabilization, Schultz–Dale response inhibition Reduced PCA in sensitized rodents	(Baruah et al. 1998)
Alginic acid	Seaweed	Histamine release inhibition, NF-κB suppression Reduced systemic and passive cutaneous anaphylaxis in mice	(Jeong et al. 2006)

### Anaphylaxis: a complex immune system underlying multiple cellular pathways

Anaphylaxis is classified as a type I hypersensitivity allergic reaction. Two major phases are involved in this kind of allergic reaction. Initially, during the sensitization or induction phase when an allergen is first encountered, antigen-presenting cells (APCs) or macrophages identify, engulf, and deliver it to naïve T cells, which will subsequently differentiate into T helper 2 (Th2) cells (Dera et al. 2020). Through the production of pro-inflammatory cytokines such as interleukin-4 (IL-4), IL-5, or IL-13, these Th2 cells transform B cells into cells that generate IgE, or plasma cells. The generated IgE attaches itself to the α-subunit of mast and basophil cells’ high-affinity IgE receptors (FcεRIs) (Chang et al. 2015; Park et al. 2020), which are the predominant effector cells in type I allergy (Li et al. 2022).

The effector phase starts when the same allergen recurs **(**Dera et al. 2020**)**. The allergen will crosslink two nearby FcεRI-bound IgE, which will set off a series of downstream signaling cascades, including those involving tyrosine kinase, protein kinase C (PKC), mitogen-activated protein kinase (MAPK) (Chang et al. 2015; Yan et al. 2024; Yoo et al. 2017), Janus kinase-signal transducer and activator of transcription (JAK/STAT), and nuclear factor κB (NF-κB) ( Liu et al. 2023). In addition, calcium influx (Yan et al. 2024) and cytoskeleton remodeling will draw in secondary cells, including neutrophils (Dera et al. 2020), which will cause degranulation (i.e., the release of histamine and β-hexosaminidase), the generation of reactive oxygen species (ROS) (Vo et al. 2020), as well as the production of various pro-inflammatory cytokines and chemokines (Barbosa et al. 2018; Chang et al. 2015; Kobayashi et al. 2015; Mwakalukwa et al. 2019; Yoo et al. 2017; Yoshioka et al. 2020).

## The most prevalent inflammatory and immunomodulatory mechanisms implicated in anaphylaxis

### FcεRI signaling pathway

An allergen’s cross-linking of IgE activates the heterotetrameric (one α, one β, and two γ subunits) FcεRI receptors, which in turn activates two protein tyrosine kinases (PTKs) belonging to the Src family, Lyn and Fyn. By recruiting spleen tyrosine kinase (Syk) to FcεRIβ immunoreceptor tyrosine-based activation motifs (ITAMs), these Lyn and Fyn activate FcεRIβ-ITAMs (Athari 2019; Li et al. 2022). PKC, rat sarcoma (Ras), phosphoinositide 3-kinase (PI3K), protein kinase B (Akt), guanosine triphosphatase (GTPase), and phospholipase Cγ (PLCγ) are among the additional signaling cascades that are activated by the Syk (Jiao et al. 2017; Lee et al. 2020; Min et al. 2021; Park et al. 2020; Yoo et al. 2017; Yoshioka et al. 2020). Activated Syk additionally activates linkers for T-cell activation (LAT) and leukocyte-specific phosphoproteins (SLP-76) that include the src homology 2 (SH2) domains.

Upon this, cytosolic adaptor molecules as glutamic acid decarboxylase 2 (Gad2), growth factor receptor bound protein 2 (Grb2), PLCγ1, and guanine exchange factors (VAV and SOS) attach to LAT, further activating the PI3K and MAPK signaling pathways (Athari 2019; Li et al. 2022).

PLCγ further lowers intracellular Ca2 + by converting phosphatidylinositol 4,5-bisphosphate (PIP2) into diacylglycerol (DAG) and inositol triphosphate (IP3) (Jo et al. 2022; Lim et al. 2023). B cell lymphoma/leukemia 10 (BCL10), mucosa-associated lymphoid tissue lymphoma translocation protein 1 (MALT1), and p38 MAPK are all activated by the activation of PKC via Ca2 + and DAG (Jo et al. 2022; Li et al. 2022). PIP2 gets transformed into phosphatidylinositol 3,4,5-triphosphate (PIP3) through the active PI3K, and PIP3 then activates ERK1/2 and JNK (via RAC and MAPK4).

Phospholipase A2 (PLA2) is activated by ERK1/2, which raises prostaglandins and leukotrienes (Jo et al. 2022). High antigen-specific IgE levels are maintained, immune cells like eosinophils are drawn to inflammatory sites, mucus production is increased, and chronic allergic inflammation that causes tissue damage and remodeling is started during the later effector phase due to the overexpression of Th2-related immune response and increased production of Th2 cytokines, such as IL-4, IL-5, and IL-13 (Barnes 2011).

### MAPK and JAK/STAT signaling pathway

These pathways are the most crucial allergy signaling pathways (Lee et al. 2020; Liu et al. 2023). A sequential activation of five protein kinases controls the MAPK signaling cascade including MAP4K, MAP3K, MAPKK, MAPK, and MAPK-activated protein kinases (MAPKAPK). Studies typically provide an explanation for MAP3K, MAP2K, and MAPK (Guo et al. 2020; Soares-Silva et al. 2016). According to their structure and function, MAPK can be divided into four groups: p38 MAPK*,* c-Jun N terminal kinase 1/2 (JNK1/2), extracellular signal-regulated kinase 1/2 (ERK1/2), and ERK5 (Li et al. 2022; Liu et al. 2023; Soares-Silva et al. 2016; Yan et al. 2024). All types of MAPK are activated by pro-inflammatory stimuli, ERK1/2 is activated by growth factors and hormones, and p38 MAPK and JNK 1/2 are activated by cellular and environmental stressors (Soares-Silva et al. 2016).

A G-protein called Ras is activated when a ligand first attaches to a receptor tyrosine kinase (RTK) in the classic activation of the ERK1/2 MAPK cascade. MAPK/ERK kinases (MEK1/2), a MAP2K sometimes referred to as MKK1/2, are activated by the serine/threonine protein kinase Raf (a MAP3K), which is recruited and activated by the Ras. These MEKs then activate ERK1/2 (a MAPK) (Cui et al. 2007; Guo et al. 2020; Soares-Silva et al. 2016; Yuan et al. 2020). The proteins upstream of the signaling cascades, such as SOS protein, Raf-1, and MEKs, are likewise given negative feedback by ERK1/2 (Guo et al. 2020). The ERK controls several transcription factors, including c-Fos, c-Jun, c-Myc, Elk-1, and ATF2, once it has been translocated into the nucleus (Guo et al. 2020). Th2 cells produce IL-4, -5, -9, and -13 via regulating the synthesis of IL-10, which is regulated by the ERK (Soares-Silva et al. 2016). In addition, Syk triggers ERK1/2, which in turn triggers the arachidonic signaling pathways and mast cell production of TNF-α, IL-2, IL-5, and IL-13. In reaction to stress or cytokines, Rho protein or tumor necrosis factor receptor-associated factor 2/3/6 (TRAF) stimulates MEKK1, SAK1, or TAK1 (a MAPK3K) They trigger the activation of MKK3 or MKK6 (a MAP2K), which in turn triggers the activation of p38 MAPK (Cui et al. 2007; Davis 2000; Soares-Silva et al. 2016; Zarubin & Han 2005).

The transcription factors ATF, NFAT, Elk-1, and HBP1 are further regulated by p38, which in turn controls the synthesis of cytokines (Zarubin & Han 2005). Th cell differentiation into Th2 cells, which generate IL-2, IFN-γ, and TNF-α/β, is boosted by the regulation of IL-12 production by the p38 MAPK (Soares-Silva et al. 2016). Numerous stressors, including cytokines (e.g., TNF and IL-1), trigger the JNK pathway via different receptors including as TNFR, GPCR, TGFBR, and TLR (Zeke et al. 2016). Rac1/Cdc42 is activated by a variety of signals, which in turn activates downstream proteins such as MLK, ASK, DLK, MEKK, and TAK. They also trigger MKK4 or MKK7, which triggers JNK **(**Cui et al. 2007; Davis 2000**)**.

In addition, DLK which is an upstream protein was provided by JNK negative feedback (Zeke et al. 2016). The JNK also activates AP-1, which controls the production of cytokines, by impacting a number of transcription factors, including c-Fos, ATF, Jun B, Jun D, and c-Jun (Cui et al. 2007; Davis 2000; Zeke et al. 2016). Elk-1, NFAT, and P53 are likewise regulated by the JNK [32]. TNF-α, IL-2, and IL-6 are activated by the JNK, while p38 MAPK stimulates IL-4 (Lee et al. 2020; Liu et al. 2023).

### JAK/STAT signaling pathway

Through the JAK/STAT signaling pathways, IL-4 and IL-13 increase the expression of pro-inflammatory genes in allergic disorders (Shankar et al. 2022). When these cytokines attach to receptors, Janus kinase (JAK) is drawn (recruited) in and the receptor dimerizes. The activated JAKs activate the receptors and attract STATs to the receptors. The active STATs separate from the receptors as homo or heterodimers, go into the nucleus, bind to DNA, and control gene expression (Hu et al. 2021; Shankar et al. 2022).

**Fig. 3:**
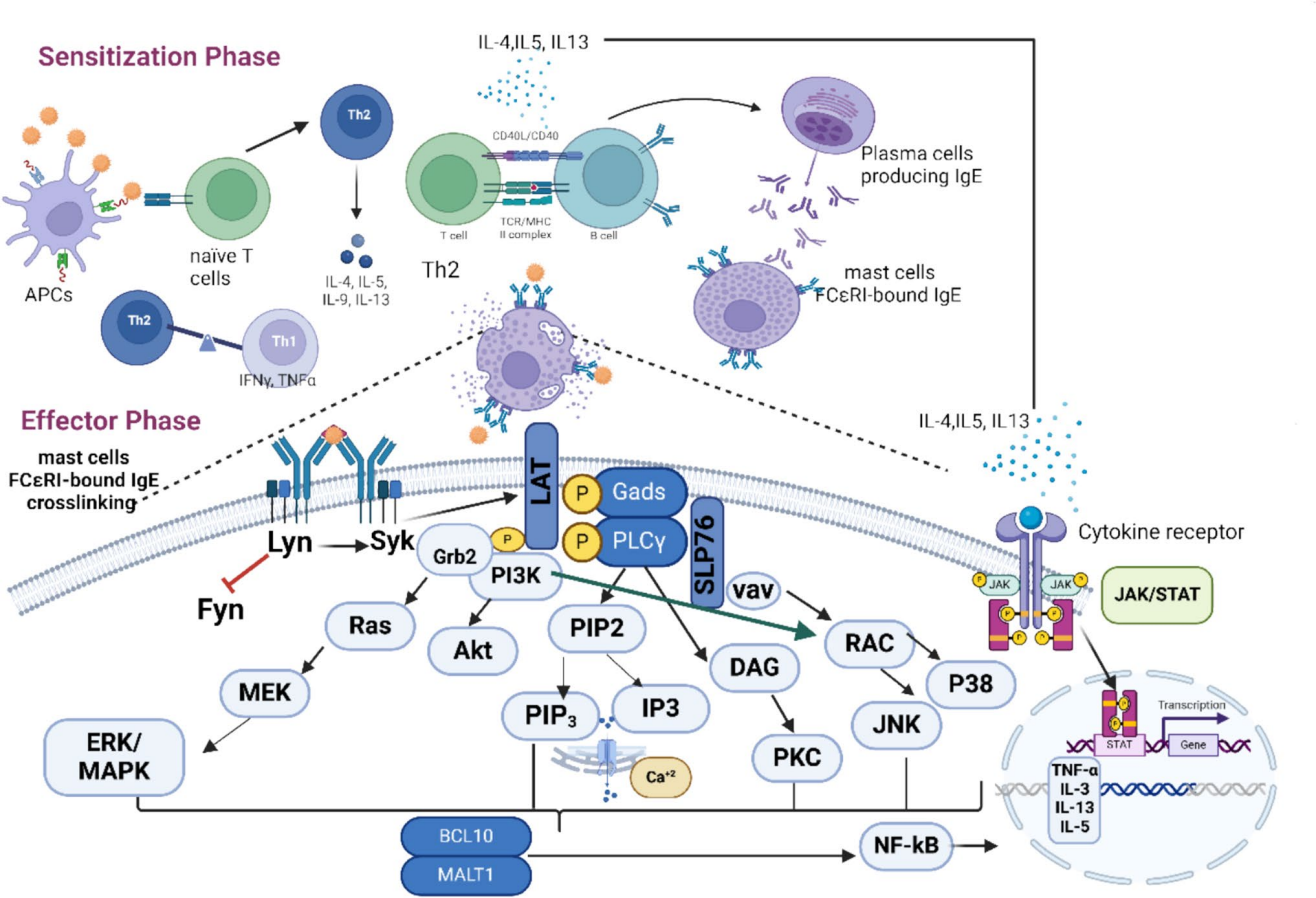
Anaphylaxis immunomodulatory–inflammatory multi-axis via sensitization and effector phases

NF-κB can be activated by MAPK (Jo et al. 2022). Trimeric forms of inactive NF-kB and an NF-kB inhibitor (IkB) are present in the cytoplasm. MEKK1 signals trigger the IkB kinase complex (IKK) (Schulze-Osthoff et al. 1997), which in turn triggers IkB (at Ser 32 and Ser 36 residues). This releases NF-kB into the nucleus, where it attaches to the promotor regions' kB binding site and triggers the activation of mediators and gene expression, including COX-2, TNF-α, and IL-1β, -6, and -8 (Chu et al. 2016; Dasiman et al. 2022; Wang et al. 2020). When the 26S proteasome ubiquitinates the activated IkB, the active NF-kB manifests as a heterodimer of the p65 and p50 subunits (Chu et al. 2016) (Fig. 3).

### Mast cell degranulation signaling pathway

Mast cells are granulocytes (Breedveld et al. 2017) that circulate around microvessels in the skin and visceral mucosa. They include heparin, histamine, and 5-hydroxytryptamine. Because of interaction between IgE antibodies and antigens linked to mast cells, the cells largely disintegrate, releasing particles and chemicals that might induce fast allergic responses in tissues. At the same time, they release a range of cytokines and help regulate the immune system. It is primarily caused by antigen-induced aggregation of FcεRI receptor molecules on the surface of mast cells, which triggers the release of inflammatory mediators from mast cells. Mast cell activation triggers the release of inflammatory mediators, which launch a signal transduction cascade. The first factor of Ca^2+^ influx in mast cells is the cross-linking of allergen and the IgE-FcRI complex, which activates phospholipase C (PLC) and produces phosphatidylinositol 4,5-diphosphate (PIP2). PIP2 produces secondary messengers such IP3 (inositol 1,4,5-triphosphate) and DAG (diacylglycerol). On the endoplasmic reticulum membrane, IP3 reacts with the IP3 receptor (IP3R) to release Ca^2+^ from the endoplasmic reticulum Ca^2+^ storage (Taylor et al. 2009). Ca^2+^ influx is predominantly induced by endoplasmic reticulum Ca^2+^ storage depletion (Michell 1975; Putney 1986). Its molecular mechanism is that the endoplasmic reticulum contains proteins STIM1 and STIM2 with EF-hand domains that may detect Ca^2+^ depletion in the calcium storage (Liou et al. 2005). Then, it migrates to the plasma membrane, where it interacts with the Orai-protein and opens the cell membrane's Ca^2+^ channel, allowing external Ca^2+^ to enter (Putney 2009). Ca2 + plays a role in the mast cell activation signaling pathway.

Ca^2+^ contributes to the mast cell activation signaling pathway. Intracellular Ca^2+^ controls cell granule movement, membrane fusion, and degranulation (Hartzell et al. 2016). FcεRI (high-affinity IgE receptor) on inactivated mast cells activates FcεRI cross-linking, leading to T-cell activation. LAT is phosphorylated in a manner determined by tyrosine-protein kinases Lyn and Syk (Zhang et al. 2002). Molecular mechanism of mast cell degranulation mainly focuses on tyrosine kinase Lyn and Fyn-dependent signal transduction pathway and increase of intracellular Ca^2+^ level (Takata et al. 1994).

Degranulation led to activation of signal transduction phospholipases PLCγ, protein kinase C, and increased calcium ions. TRPC1 channels also promoted Ca^2+^ inflow (Svetlov et al. 1996). The activation of the RAS-RAF-MAPK pathway produces eicosanoids (including leukotrienes C4 and prostaglandin D2) as well as cytokines. InsP3R (Inositol Triphosphate Receptor), a membrane glycoprotein complex triggered by InsP3, functions as a calcium channel (Hagar et al. 1998), while STIM (Stromal interaction molecules) is a calcium receptor on the endoplasmic reticulum (Wang et al. 2021a, b). Therefore, blocking the binding of FcRI and IgE effectively inhibits allergic reactions.

### Oxidative stress and mast cell degranulation

Recent research has demonstrated immediate hypersensitivity responses caused by the activation of Mas-related G protein coupled receptor member X2 (MRGPRX2) expressed in mast cells. The binding of these chemicals to the MRGPRX2 receptor activates cytosolic G proteins, elevates intracellular calcium concentrations, and eventually causes the exocytosis of anaphylactic mediators (Subramanian et al. 2016). There is a relationship between mast cell degranulation and mitochondrial respiratory complex chain activity, which causes oxidative phosphorylation. Usually, mast cell activation is linked with increased mitochondrial respiration. Thus, IgE-mediated activation increased oxygen consumption rate by 60% (Buttgereit et al. 2022).

As a result, inhibiting mitochondrial respiratory complex chain lowered both IgE- and non-IgE-mediated mast cell degranulation. Inhibiting complexes, I and III inhibited IgE-mediated mast cell degranulation (Suzuki et al. 2005; Takekawa et al. 2012). Enzymatic reactions occur in cells and involve the transfer of energy, electrons, or protons. Some molecules, such as oxygen or nitrogen, can acquire or transfer electrons, causing them to become unpaired. This new molecule is known as a “free radical” because it may interact with other, more stable molecules. Reactive oxygen species (ROS) and reactive nitrogen species (RNS) contribute to physiological activities required for appropriate cellular function, as well as pathological processes that affect energy generation, apoptosis, or mitophagy. Oxidative stress is characterized as an imbalance in ROS generation and neutralization by antioxidant mechanisms. IgE activation of mast cells resulted in elevated intracellular ROS levels and degranulation (Collaco et al. 2006; Tagen et al. 2009).

Following allergen stimulation, intracellular ROS generation in mast cells increased in a time and allergen dose-dependent manner before plateau (Yasui et al. 2007). Allergen stimulation via IgE receptor FcεRI activates Lyn, Fyn, and Syk kinases, leading to activation of PLCγ and PI3K. PLCγ creates DAG and IP3, resulting in calcium-dependent degranulation. IP3 interacts to its receptors IP3R on the endoplasmic reticulum, causing Ca2 + release. PI3K stimulates ROS generation via NOX action. MRGPRX2 activation enhances PI3K and PLCγ activation. Increased intracellular Ca2 + causes granule exocytosis. ROS modulates mast cell degranulation via Ca2 + efflux from the endoplasmic reticulum and extracellular influx.

mtROS controls PKC phosphorylation, resulting in degranulation. Mast cell activation causes mitochondrial fragmentation, morphological alterations, and the relocation of mitochondria from the perinuclear area to the cell surface. Phosphorylation of the two transcriptional factors STAT3 and MITF causes mitochondrial respiration, ATP generation, and mitochondrial ROS, which promotes mast cell degranulation.

**Fig. 4:**
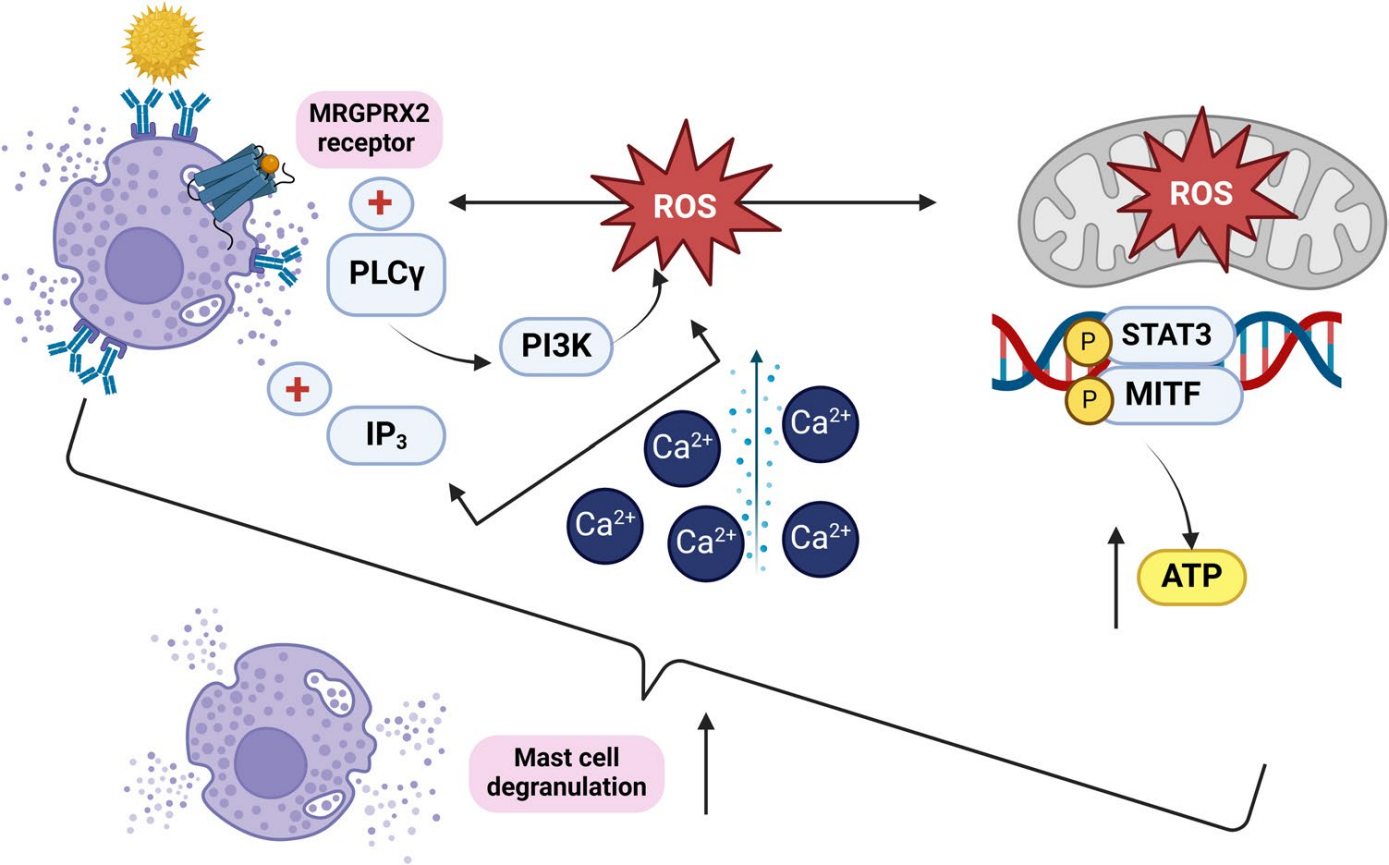
Relation between oxidative stress and mast cell degranulation

Thus, ROS play an important role in mast cell control, including activation and degranulation. In conclusion, a recent study found that antigen activation of mast cells causes enhanced ROS generation from NADPH oxidases and the mitochondrial respiratory chain. In-vitro antioxidant therapy of mast cells seems to prevent degranulation by lowering ROS levels, but further research is needed to validate this. Exposure to pro-oxidant environmental circumstances, such as air pollution, appears to promote mast cell degranulation via boosting ROS production (Piotin et al. 2024) (Fig. 4).

**Fig. 5:**
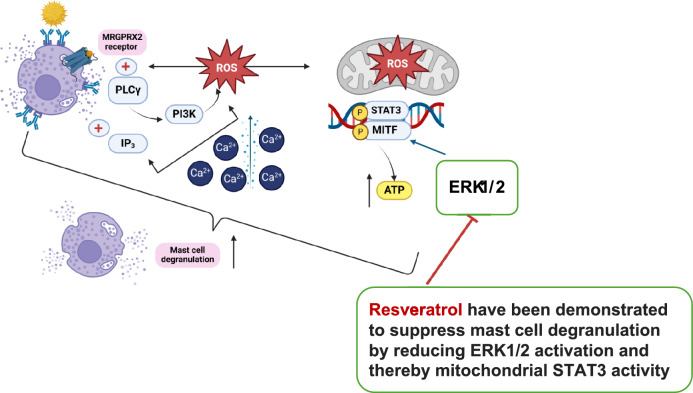
Polyphenols defeat oxidative stress action towards mast cell degranulation

Some natural compounds, such as the polyphenol and resveratrol, have been demonstrated to suppress mast cell degranulation by reducing ERK1/2 activation and thereby mitochondrial STAT3 activity (Paradis et al. 2024). Furthermore, after mast cell activation, PIAS3, the major endogenous inhibitor of STAT3 found in mitochondria, rose and worked as a regulator of the mitochondrial allergic response (Erlich et al. 2014) (Fig. 5).

## Mechanisms of action of natural products in treatment of anaphylaxis

Through a variety of molecular pathways, it has recently been shown that a wide range of natural compounds, such as flavonoids, terpenes, phenols, saponins, alkaloids, coumarins, lignans, and quinones, might reduce the symptoms of hypersensitivity type I reactions, including anaphylactic reactions.

**Fig. 6:**
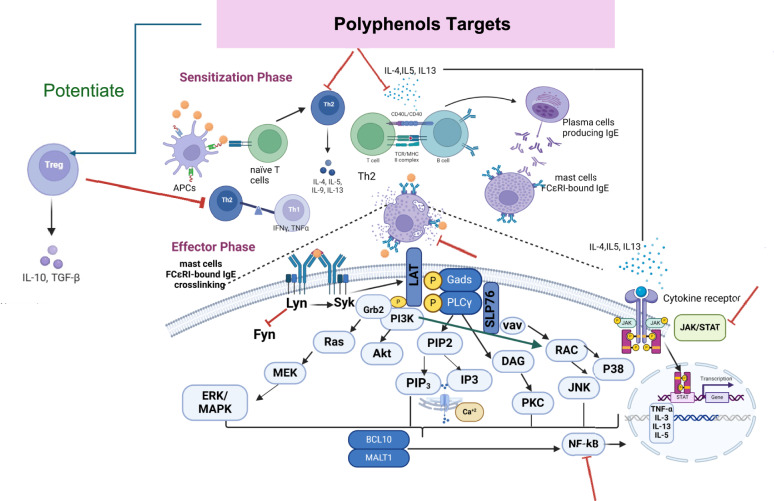
Summary of the polyphenols most common anti-anaphylactic effect among anaphylaxis pathways

One of the most well-known natural bioactive substances with well-established anti-inflammatory, antioxidant, and immunomodulatory qualities is dietary polyphenols, which have been the subject of numerous studies. Polyphenols are a valuable and promising dietary intervention in the prevention and treatment of allergic diseases because of their safety profile, broad distribution in plants, frequent inclusion in the daily diet, and a wide range of bioactivity, including immunomodulatory and anti-inflammatory qualities (Eseberri et al. 2022; Rakha et al. 2022; Rana et al. 2022). Thus, in response to the increasing need for the creation of novel therapeutic and preventative options based on natural products, polyphenols have attracted a lot of scientific attention and been the focus of in-depth study in recent years (Kumari et al. 2023; Wu et al. 2023; Zeng et al. 2023a, b). Quercetin, curcumin, resveratrol, catechins, and many other polyphenols may reduce allergic inflammation, relieve food allergy, asthma, and allergic rhinitis symptoms, and stop the onset of an allergic immunological response. It is yet unknown and requires clarification of what precise molecular and cellular processes polyphenols may use to prevent and treat allergy disorders. Yet, it is hypothesized that polyphenols’ advantageous anti-allergic action is linked to their impact on primary targets such as interaction with allergic proteins and decrease in their allergenicity, modification of the systemic and local immune response (Fig. 6).

### Interaction with allergic proteins and decrease in their allergenicity

#### Allergic protein modification

In fact, it has been discovered that certain polyphenols can reduce allergenicity by changing the secondary and tertiary structures of proteins, which alters the conformational epitopes of the allergen, and by masking the linear epitopes of the allergen by conjugation with nucleophilic amino acids (Liu et al. 2021a, b; Yan et al. 2023; Zhang et al. 2021; Zhou et al. 2024). Numerous investigations concerning the phytochemical alteration of a main allergen in cow’s milk β-lactoglobulin (β-LG) have shown that covalent conjugation with different polyphenols, such as rutin, ferulic acid, caffeic acid, epigallocatechin (EGCG), and chlorogenic acid, results in conformational changes that cause proteins to become more unfolded, which is correlated with a decreased ability of IgG/IgE to bind (Deng et al. 2023; Liu et al. 2021a, b; Wang et al. 2023; Wu et al. 2018; Xu et al. 2019; Xue et al. 2023). Moreover, a number of flavonoids, including EGCG, naringenin, myricetin, kaempferol, and quercetin, can also reduce the allergenicity of β-lactoglobulin through non-covalent interactions. EGCG demonstrated the strongest inhibitory potency on β-LG antigenicity, resulting in a 73% decrease in IgE binding ability (Pu et al. 2021).

The ovalbumin allergenicity was evaluated in vitro as the ability to cause effector cell degranulation and in vivo as the severity of the allergic immune response and symptom score (T. Zhang et al. 2020). Likewise, the covalent conjugation between quercetin and ovalbumin altered the secondary and tertiary conformation of the protein, resulting in a less folded structure and decreased allergen stability. Furthermore, following covalent and non-covalent binding with quercetin, the spectrometric structural analysis of allergens belonging to the profilin family revealed a 40% reduction in the α-helical structures in the conjugates. This, in conjunction with the antigenic epitopes being obscured, led to a significantly reduced level of allergenicity (Zhou et al. 2023).

According to recent reports, covalent interactions with CA, EGCG, and polyphenols isolated from the algae Sargassum fusiforme can cause conformational changes in shrimp tropomyosin's structure. These alterations can significantly reduce allergenicity, which in turn reduces shrimp-induced allergic symptoms in vivo (Lv et al. 2021; Zhao et al. 2023).

#### Boosting the allergen’s digestibility

For example, experimental investigations have shown that covalent attachment of EGCG and CA to peanut proteins reduces allergenicity dramatically, not only by modifying linear and conformational epitopes, but also by enhancing peanut allergen digestion.

In vitro and in a food allergy mouse model, peanut protein was found to have lesser allergenicity, resulting in fewer symptoms, mast cell frequency, and intestinal damage (Bansode et al. 2019; He et al. 2023, 2020).

Five major apple polyphenols (epicatechin, phlorizin, rutin, chlorogenic acid, and catechin) showed similar effects on simulated gastric digestion and the spatial structure of Ara h 1 peanut protein. Epicatechin was found to have the strongest inhibitory effect on peanut allergy (Sun et al. 2023).

Furthermore, the IgE/IgG binding capacity was impacted by the covalent binding of wheat gliadin with luteolin and chlorogenic acid, which impacted the protein conformation and gave it a more ordered structure. This also greatly enhanced the thermal stability and in-vitro digestibility of allergic proteins (Yang et al. 2023a, b; Zhang et al. 2022a, b).

#### Polyphenols may reduce allergen load by causing protein aggregation and cross-linking

Possibly due to the loss of reactive allergens and reduced accessibility of reactive epitopes (Bessa et al. 2021; Zhang et al. 2021; Zhou et al. 2024). Meanwhile, Polyphenols’ ability to form cross-linked protein polymers allows allergen binding to be efficient even when the number of polyphenol molecules is less than the number of allergen reaction sites, and the resulting polyphenol–allergen complexes are more stable and thus more effective (Bessa et al. 2021; Pi et al. 2023; Zhou et al. 2024). This effect was clearly demonstrated in a large number of studies examining the structural and functional features of several soybean globulins following covalent interaction with polyphenols such as EGCG, chlorogenic acid, caffeic acid, gallic acid, and tannic acid (Li et al. 2023; Lin et al. 2022; Pi et al. 2023; Zhou et al. 2020). In all cases, the formation of polyphenol-soybean globulin conjugate and cross-linking of soybean proteins resulted in structural changes that concealed or destroyed allergen epitopes, as well as increased UV absorption and protein digestibility, resulting in reduced IgE binding activity and histamine release in vitro (Li et al. 2023; Lin et al. 2022; Pi et al. 2023; Zhou et al. 2020).

It is interesting to note that studies using the mouse model of allergy showed that covalently conjugating the soy 11S protein with EGCG and chlorogenic acid can effectively induce the development of oral tolerance to soy allergen in addition to lowering the protein’s allergenicity and easing allergy symptoms (Li et al. 2023). Combining all the aforementioned information, dietary polyphenols have a significant potential to decrease food allergenicity; as a result, they may be helpful in creating hypoallergenic meals that may lessen the symptoms of food allergies or even stop them from occurring by fostering tolerance.

### Immunomodulatory effect

Polyphenols have been shown to have both stimulatory and inhibitory effects at two crucial stages, during the sensitization and effector phases of allergic disease, according to evidence gathered from in-vitro and in-vivo studies (del Cornò et al. 2016; Haftcheshmeh et al. 2022; Kumari et al. 2023; Magrone et al. 2020; Mlcek et al. 2016; Mwakalukwa et al. 2019; Rakha et al. 2022; Shaik et al. 2018; Shakoor et al. 2021; Wu et al. 2023; Zeng et al. 2023a, b; Zhang et al. 2022a, b).

#### Sensitization phase

Dendritic cells (DCs) present the entering allergen to naïve CD4 + T cells in draining lymph nodes as the initial step in the sensitization phase. This causes the naïve CD4 + T cells to differentiate into allergen-specific Th2 cells that produce proallergic cytokines (IL-4, IL-5, IL-9, and IL-13) (Humeniuk et al. 2017). By influencing DC differentiation, maturation, and the ability to trigger T-cell differentiation into allergy type Th2 cells, some polyphenol groups have been shown to obstruct the antigen presentation pathway (del Cornò et al. 2016). Resveratrol affect how human DC differentiate from monocytes and prevent DC maturation, which results in the production of an immature phenotype (Buttari et al. 2013; Švajger et al. 2010). Several polyphenols, including quercetin, curcumin, fisetin, silibinin, isoflavones, and blackberry polyphenols, have been shown to inhibit the phenotypic and functional development of DC generated from murine bone marrow. Furthermore, these substances suppress the expression of major histocompatibility complex (MHC) class II and co-stimulatory molecules (CD83, CD80, and CD86) on the surface of DCs, which impedes effective antigen presentation (Dai et al. 2007; Huang et al. 2010; Liu et al. 2010; Yoneyama et al. 2008; Yoon et al. 2006). In addition to influencing DC differentiation and reducing antigen absorption activity, other polyphenols, EGCG, and apigenin have also been shown to induce apoptosis in DC-precursors and immature DCs (Kang et al. 2009; Yoneyama et al. 2008).

In addition, the next significant event in the sensitization phase, naïve CD4 + T cell priming, can be regulated by polyphenols. Indeed, it has been demonstrated that kaempferol and lycoricidine disrupt TCR-mediated signaling cascades, therefore, preventing naïve CD4 + T cells from activating and differentiating into Th2 effector cells (Lee & Jeong 2021; Lee et al. 2021). Apart from DCs presenting allergens, cytokines such as TSLP, IL-25, and IL-33, which are released by epithelial cells lining barrier sites in response to food and aero-allergens, are crucial in the allergic sensitization phase because they activate DCs and innate lymphoid cells type 2 (ILC2) and stimulate the growth of Th2 cells (Hammad & Lambrecht 2015; Pasha et al. 2019).

By generating IL-4, IL-13, and IL-5 during the early stages of antigen sensitization, ILC2 are also crucial for promoting the Th2 immune response (Zheng et al. 2021). In human keratinocyte models of atopic dermatitis (AD) as well as AD-like mice models, many polyphenols, including quercetin, curcumin, and baicalin, have been found to inhibit the production and secretion of TSLP and IL-33 (Beken et al. 2020; Sharma et al. 2020; Wang et al. 2022). Resveratrol and naringenin, two other polyphenols have the ability suppress TSLP synthesis and mRNA expression in human mast cell lines (Moon et al. 2011, 2021). In experimental models of allergic airway inflammation, quercetin was also shown to have a modulatory influence on epithelium-derived cytokines, as evidenced by its considerable reduction of TSLP expression in lung tissue and IL-25, IL-33, and TSLP levels in BAL (Caglayan Sozmen et al. 2016).

In the mouse model of allergic rhinitis and asthma, it was recently reported that *Fallopia japonica*, also known as Asian knotweed, a traditional medicinal herb rich in polyphenols such as resveratrol and flavones, targets the IL-33/TSLP signaling pathway and significantly lowers these cytokine levels in both nasal and bronchoalveolar lavage fluid (Jin et al. 2023).

Although polyphenols may have an impact on B cell recruitment, maturation, and function, this effect has not yet been fully explored and documented (Bessa et al. 2021; Shakoor et al. 2021; Singh et al. 2011). However, several polyphenols, including curcumin, rosmarinic acid, quercetin, ferulic acid, tea catechins (EGCG, ellagitannins, and gallic acid), and red grape polyphenols, have been shown in in-vitro and in-vivo studies to have the ability to inhibit the production of antigen-specific IgE in a dose- and time-dependent manner (Acar et al. 2016; Liang et al. 2020; Magrone et al. 2020).

Dihydromyricetin, a naturally occurring flavonoid, was used to demonstrate the modulatory effect of polyphenols. It successfully suppressed the sensitization phase by lowering the number of B cells and their production of antigen-specific IgE and by blocking the FcεRI–IgE interaction (Zhang et al. 2019). Likewise, tea catechins and phlorotannins (such as dieckol and eckol) may interact with FcεRI by directly binding to the α chain, preventing antigen-specific IgE from binding to FcεRI and reducing the mast cell sensitization phase (Kim et al. 2020; Li et al. 2021; Sugiura et al. 2021).

By hampering the expression of the FcεRI receptor, which is essential for the long-term sensitization of mast cells and their subsequent degranulation during the effector phase, it has also been demonstrated that phlorotannins, saponins, catechins, quercetin, kaempferol, and resveratrol may help to attenuate the allergic reaction (Li et al. 2021; Nagata et al. 2023; Zhang et al. 2022a, b).

#### Effector phase

Whenever the same allergen is encountered again during the effector phase, IgE bound to FcεRI on the surface of mast cells and basophils cross-links, activating and degranulating them and releasing reactive mediators that cause an acute systemic allergic response (Pasha et al. 2019). The ways in which polyphenols may modulate mast cells, which are important effector cells of the allergic reaction, have been the subject of several recent in-vitro and in-vivo investigations (Rakha et al. 2022; Wu et al. 2023; Zhang et al. 2022a, b). Apart from the previously mentioned effects on FcεRI receptor expression and FcεRI–IgE binding, various polyphenols, resveratrol, quercetin, and procyanidins from apple extract or cinnamon, can inhibit mast cell activation by preventing allergens from cross-linking IgE on the cell surface (Civelek et al. 2022; Kandhare et al. 2017; Shaik et al. 2018). Furthermore, some polyphenols, such as quercetin, phlorotannins, luteolin, and myricetin, have been shown to downregulate the expression of calcium channel proteins and inhibit calcium influx and intracellular calcium elevation, which are required for mast cell degranulation (Mwakalukwa et al. 2019; Park 2020; Sugiura et al. 2021; Vo et al. 2020). This has been shown to stabilize mast cell membranes and thereby suppress their degranulation. The release of histamine and β-hexosaminidase, which are indicators to assess the degree of mast cell degranulation, was in fact markedly inhibited by these phenolic substances, curcumin, EGCG, rosmarinic acid, and resveratrol (Barbosa et al. 2019; Civelek et al. 2022; Kong et al. 2020; Magrone et al. 2020; Mwakalukwa et al. 2019; Vo et al. 2020).

Furthermore, polyphenols have been shown to be a strong suppressor of the MAPK and NF-κB signaling pathways, as well as the FcεRI-mediated protein kinases (Syk, Lyn, PLCγ, and PKC) signaling cascade, which are essential for the allergic reaction. This has been shown to attenuate the production of lipid mediators (prostaglandin D2, leukotrienes) and pro-inflammatory cytokines (IL-4, TNF-α) (Civelek et al. 2022; Crozier et al. 2023; Hwang et al. 2019; Vo et al. 2020; Yousef et al. 2020; Zeng et al. 2023a, b). The anti-allergic potential of stingless bee honey (Kelulut honey) was interestingly confirmed via the mechanism of mast cell activation and degranulation. However, the inhibitory effect was solely dependent on the botanical source of honey, as it was only indicated in the case of honey that was rich in polyphenols and derived from rubber and bamboo trees, while honey that was inadequate in polyphenols and derived from noni and mango did not exhibit such anti-allergic action (Yong et al. 2023).

The immunomodulatory effects of polyphenols at several critical stages of the effector phase, such as inhibition of Th2 differentiation, downregulation of Th2-related cytokine production, reduction of inflammatory cell infiltration, and consequently, suppression of allergic inflammation, have been confirmed by numerous experimental studies employing cellular and animal models.

Most significantly, polyphenols were discovered to successfully resolve the Th1/Th2 imbalance by inhibiting the overexpression of Th2-mediated immune responses and upregulating the Th1 pathways **(**Rakha et al. 2022; Shakoor et al. 2021; Singh et al. 2011**)**. Curcumin has demonstrated anti-allergic effects in a number of models of allergic diseases, including lowering Th2 cell activity and proliferation, lowering IL-4, IL-5, and IL-13 secretion, preventing macrophage, monocyte, neutrophil, and eosinophil activation and infiltration into inflammatory sites, and shifting the Th1/Th2 response toward the Th1 phenotype **(**Chauhan et al. 2018; Shahgordi et al. 2020; Shahid et al. 2019; Sharma et al. 2020**)**. By reducing the synthesis of IL-4, Il-5, and Il-13 in the serum and bronchoalveolar lavage fluid (BALF), kaempferol and rosmarinic acid, with particular consideration paid to asthma models, attenuated airway inflammation. They also successfully decreased the recruitment of eosinophils into lung tissues, airway hyperresponsiveness, and mucus production **(**Liang et al. 2020; Molitorisova et al. 2021; Shakeri et al. 2019**)**. Flavonoids such as quercetin, isoquercetin, myricetin, and luteolin have been shown in studies in a mouse model of allergic rhinitis to reduce inflammation of the nasal mucosa by promoting the Th1 pathway and preserving the Th1/TH2 balance in addition to inhibiting Th2 cell differentiation and cytokine secretion **(**Dong et al. 2021; Feng et al. 2020; Hwang et al. 2018; Ke et al. 2023; Shi et al. 2023**)**. Furthermore, in mouse models of food allergy, quercetin and tea catechins (gallic acid and ellagitannins) have been demonstrated to be potent inhibitors of the ovalbumin (OVA)-induced allergic response, promoting immune tolerance through Th1/Th2 modulation and induction of regulatory T cells (Treg) **(**Feng et al. 2020; Mine et al. 2020**)**. Indeed, disruption of the equilibrium between Th17/Treg cells, in addition to Th1/Th2 dysregulation, plays an essential role in the development and progression of chronic allergic inflammation by contributing to the breakdown of immunological tolerance (Boonpiyathad et al. 2020; Tortola et al. 2020). By increasing the amount of Tregs and reestablishing the balance between Th17/Treg, several flavonoids, including quercetin, luteolin, cyanidin, and baicalin, have been shown in recent experimental trials to have an anti-allergic impact (Ke et al. 2023; Li et al. 2020; Liu et al. 2017; Yang et al. 2023a, b).

In a murine model of asthma, curcumin also had a modulatory impact on the Th17/Treg imbalance, considerably boosting the frequency of Treg subtypes while successfully decreasing Th17 cell differentiation (Chen et al. 2018; Ma et al. 2013; Wu et al. 2020). In conclusion, a wealth of evidence from animal models and in-vitro experiments suggests that polyphenols may be able to prevent the onset of allergic diseases by modifying the process of allergic sensitization. In addition, their effects on allergy effector cells upon re-exposure may represent a novel therapeutic approach.

## Conclusions and future directions

This article offers a thorough examination of the molecular mechanisms through which natural compounds exert therapeutic effects on anaphylaxis. In general, these natural compounds demonstrate distinct benefits in managing allergic inflammation, and most are safe and easily accessible. However, it is essential that these natural compounds undergo further evaluation through rigorous and extensive clinical studies. There is a need for greater emphasis on clinical translational research to improve the quality of evidence from clinical studies. There is a notable lack of clinical trials specifically evaluating natural products for their efficacy in managing anaphylaxis. Although many natural compounds have anti-inflammatory and anti-allergic effects and have been investigated in preclinical settings, animal models and in-vitro studies, their direct use in anaphylaxis has not been thoroughly tested in human clinical trials. The complexity and urgency of anaphylactic reactions, which require rapid intervention, making it challenging to design controlled studies, contributing in part to this disparity. Furthermore, the field lacks standardized protocols for testing natural products in severe allergic conditions, and ethical concerns further limit such trials. Rather than acute anaphylaxis, current data are mostly derived from studies on related disorders including allergic rhinitis, asthma, and atopic dermatitis. For natural products to be integrated into anaphylaxis management, rigorous, large-scale clinical trials are essential to validate their safety, efficacy, and mechanism of action in this context. In addition, the specific targets of natural products in treating anaphylaxis remain unclear, and multi-omics approaches may help identify these functional targets. Many natural products exhibit poor absorption, distribution, metabolism, excretion (ADME) properties, and low water solubility. This highlights the need for chemical modifications or the use of nano-based drug delivery systems to enhance their effectiveness. Overall, addressing these limitations through focused research could significantly advance the therapeutic application of natural compounds in managing allergic conditions.

## Data Availability

No datasets were generated or analyzed during the current study.
